# Oriented catalysis through chaos: high-entropy spinels in heterogeneous reactions

**DOI:** 10.1039/d4sc05539j

**Published:** 2024-12-27

**Authors:** Yalan Mo, Xiaohong Guan, Shaobin Wang, Xiaoguang Duan

**Affiliations:** a School of Chemical Engineering, The University of Adelaide Adelaide SA 5005 Australia shaobin.wang@adelaide.edu.au xiaoguang.duan@adelaide.edu.au; b School of Ecological and Environmental Science, East China Normal University Shanghai 200241 China

## Abstract

High-entropy spinel (HES) compounds, as a typical class of high-entropy materials (HEMs), represent a novel frontier in the search for next-generation catalysts. Their unique blend of high entropy, compositional diversity, and structural complexity offers unprecedented opportunities to tailor catalyst properties for enhanced performance (*i.e.*, activity, selectivity, and stability) in heterogeneous reactions. However, there is a gap in a critical review of the catalytic applications of HESs, especially focusing on an in-depth discussion of the structure–property–performance relationships. Therefore, this review aims to provide a comprehensive overview of the development of HESs in catalysis, including definition, structural features, synthesis, characterization, and catalytic regimes. The relationships between the unique structure, favorable properties, and improved performance of HES-driven catalysis are highlighted. Finally, an outlook is presented which provides guidance for unveiling the complexities of HESs and advancing the field toward the rational design of efficient energy and environmental materials.

## Abstract 2

This review provides an overview of the advances in high entropy spinel oxides in diverse catalytic reactions and highlights the intrinsic structure–property–performance relationships.

## Graphical abstract

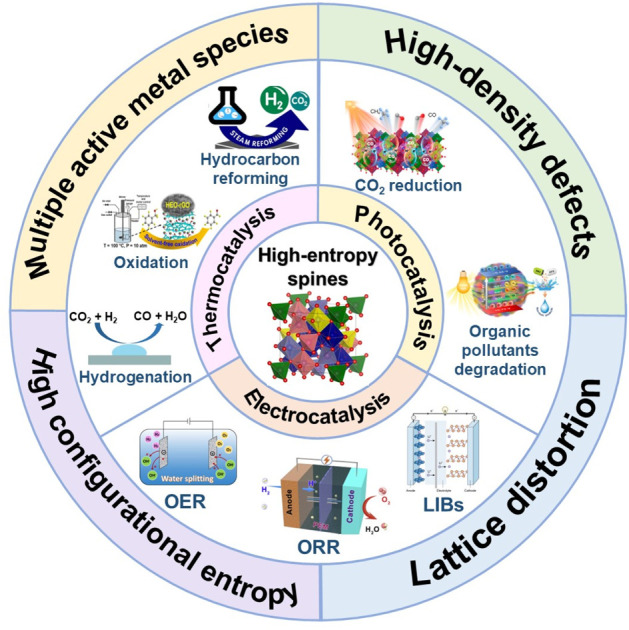

## 1. Introduction

Heterogeneous catalysis is the cornerstone of the modern chemical industry, playing a pivotal role in a wide range of industrial processes, including energy conversion, environmental remediation, and fine chemical production.^1^ Traditional catalysts, typically composed of noble metals (*e.g.*, Pt, Ir, and Ru) or transition metal oxides (*e.g.*, Co_3_O_4_, MnO_2_, and Fe_3_O_4_), have been widely used due to their exceptional catalytic properties.^2,3^ However, these materials face limitations such as high cost, scarcity, and deactivation under harsh conditions.^4–6^ Thus, it is necessary to design and develop cost-effective and high-performance catalysts to satisfy the growing demands for practical applications.

High-entropy materials (HEMs) are new favorite catalysts featuring multielement composition and complex atomic configurations in the field of catalysis. From high-entropy alloys (HEAs) as the first-reported HEMs, a broader range of HEMs, including high-entropy oxides (HEOs) and other high-entropy ceramics (HECs), have been explored.^7–13^ Among them, high-entropy spinels (HESs) have attracted significant attention due to their notable catalytic properties stemming from the transition metal components, high electrical conductivity, structural robustness, and stability.^14–16^ Unlike the rock-salt structure, the spinel structure offers unique opportunities for multicomponent systems due to its large and complex unit cell, consisting of 32 anion sites surrounded by 24 cations arranged in both octahedral and tetrahedral cages.^17–19^ Such a spinel structure endows HESs with remarkable compositional versatility, enabling fine-tuning of their structural, electronic, and surface properties. This flexibility makes HESs highly versatile and efficient catalysts.

Recent studies have demonstrated the excellent performance of HESs in various catalytic processes, such as the oxygen evolution reaction (OER) and oxygen reduction reaction (ORR) and in Li-ion batteries (LIBs), highlighting their potential as next-generation catalysts.^20–25^ However, the full potential of HESs in catalytic applications has largely remained untapped due to limited knowledge of the relationships between the structure, properties, and catalytic performance of HESs. The complexity of HESs, arising from the vast compositional space and intricate atomic arrangements, poses significant challenges in elucidating the mechanisms underlying their catalytic behaviour. It is essential to employ advanced experimental and computational methods to systematically study the intricate structure–property–activity relationships of HESs in catalysis. In addition, understanding the unique structural attributes of HESs, such as multiple active metal sites, defect structures, and high configurational entropy to their catalytic activity, can inform the rational design of more efficient and selective catalysts.

This review aims to provide a comprehensive overview of the current research on HESs in heterogeneous catalysis, highlighting key advancements and identifying critical knowledge gaps. Firstly, the fundamentals of HESs are introduced, including a historical overview, definition and features, synthesis methods, and characterisation techniques. Secondly, the research progress of HESs in thermocatalysis, electrocatalysis, and photocatalysis is reviewed. More importantly, the structure–property–performance relationships of HESs and catalytic reaction mechanisms are highlighted and discussed. At the end, challenges and opportunities are discussed to outline potential future directions in developing next-generation HESs for sustainable heterogeneous catalysis.

## 2. High-entropy spinels

### 2.1 A historic overview

HESs originated from the exploration of an alloy, a metallic substance containing two or more metal or non-metal elements.^26^ Traditional alloys typically consist of a primary element melted with one or two additional elements in minor quantities, limiting their elemental diversity and broader applications.^27^ In 2004, Cantor *et al.* and Yeh *et al.* introduced “high-entropy alloys”, featuring multiple principal elements in nearly equal atomic proportions, to enhance the diversity and properties of metal alloys.^7,28^ Inspired by the research on HEAs, Rost *et al.* extended the high-entropy concept to five-component oxides in 2015, preparing the first HEOs with a rock-salt structure.^8^ Compared with the simple face-centered cubic (FCC),^29^ body-centered cubic (BCC),^30^ and hexagonal close-packed (HECP) structures in HEAs,^31^ various crystal structures of HEOs have been demonstrated, including rock-salt,^32^ perovskite,^33^ spinel,^34^ fluorite,^35^ bixbyite,^36^ pyrochlore,^37^ and layered O3-type structures.^38^

**Fig. 1:**
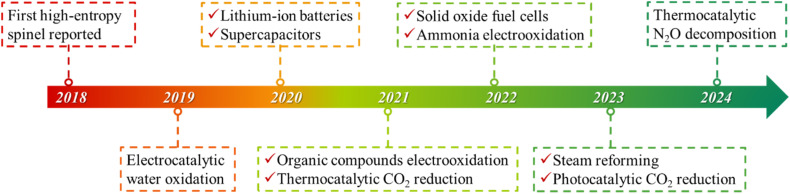
Timeline of major developments in HESs and their key applications.

Rock-salt HEOs which contain only one Wyckoff site for cations have been mostly studied due to their easy formation. With the further development of HEOs, spinel- and perovskite-structured HEOs with multiple Wyckoff sites have attracted increasing attention, because they have more diversified atomic arrangements and thus possess greater flexibility in composition and property tuning.^39–42^ In 2018, Dąbrowa *et al.*, for the first time, synthesized the single-phase spinel (Co, Cr, Fe, Mn, Ni)_3_O_4_ HEO *via* a solid-state reaction method.^43^ Since then, numerous HESs have been reported and studied in a wide range of catalytic applications, including electrochemical water splitting,^44^ anode materials for LIBs,^45^ supercapacitor electrode materials,^46^ electrooxidation of organic compounds,^47^ thermocatalytic CO_2_ reduction,^48^ cathode materials for solid oxide fuel cells,^49^ steam reforming,^50^*etc.*^16,22,51^ A timeline of major developments in HESs and some key applications is illustrated in Fig. 1.

### 2.2 Definition and features

#### 2.2.1 Definition

The concept of HESs is based on HEAs. Despite some controversy over the definition of HEAs, the universal recognition from the perspectives of composition and entropy has been widely accepted. For the composition-based definition, HEAs are generally prepared from at least five principal elements with atomic concentrations from 5% to 35%.^7^ For the entropy-based definition, HEAs are described using the mixing configuration entropy (Δ*S*_mix_), which is defined as the degree of disorder of a system. The Δ*S*_mix_ of a typical HEA is expressed as follows:^27^1
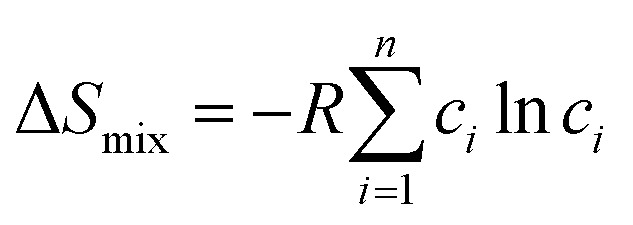
where *R* is the gas constant, *c*_*i*_ is the molar fraction of each elemental component, and *n* is the number of elements. The value for Δ*S*_mix_ can be maximized when the proportions of each constituent are equal, represented by the following expression:

2 Δ*S*_mix_ = −*R* ln *n*

In this definition, a HEA is a material in which the ideal configurational entropy equals or exceeds the value of five elements in equiatomic proportions, specifically Δ*S*_mix_ ≥ 1.61*R*; materials with Δ*S*_mix_ ≤0.69*R* are considered “low entropy”, and those with 1.61*R* > Δ*S*_mix_ > 0.69*R* are “medium entropy”.^52,53^ Other studies considered 1.5*R* to be the boundary between medium entropy and high entropy and ≤ 1.0*R* to be the low entropy cutoff.^54,55^

As HESs contain both cations and anions, the Δ*S*_mix_ was further extended to^56^3
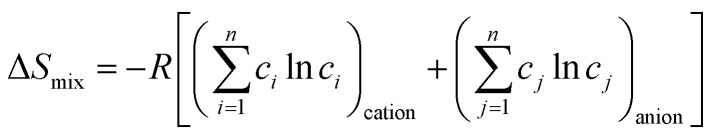
where *c*_*i*_ and *c*_*j*_ are the molar fractions of anions and cations, respectively.

It should be noted that the terms “high entropy”, “multicomponent”, and “compositionally complex” are not synonymous. A material being “multicomponent” or “compositionally complex” does not automatically indicate high entropy, as configurational entropy depends on not only the number of components but also their proportions. In addition, although configurational entropy is typically considered the main contributor to the total entropy of a solid, nonconfigurational contributions (such as vibrational, electronic, or magnetic entropy) may dominate the thermodynamics in some cases and should be considered.^57^

#### 2.2.2 Features

**Fig. 2:**
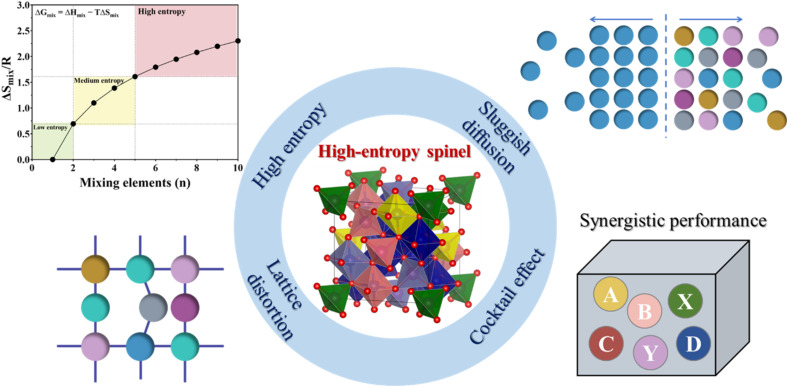
Schematic illustration of the unique features of HESs.

In typical normal spinels, AB_2_X_4_, metal A occupies the centers of tetrahedral sites, metal B occupies octahedral sites, and the anion (*e.g.*, O^2−^) sits at the polyhedral vertices.^58,59^ The anionic X site typically adopts the −2 valence state, and the cationic A species can exist in the +2 or +4 valence state, while cation B is present in the +3 or +2 oxidation state. Spinels can also exist with inverse structures, where half of the B cations occupy the centers of the tetrahedral sites, whereas A and the remaining half of the B occupy the octahedral sites, which can be described as B(AB)X_4_. In many cases, the actual configurational structure is between normal and inverse, and the degree of inversion (*λ*) is the parameter employed to describe them according to A_1−*λ*_B_*λ*_(A_*λ*_B_2−*λ*_)X_4_ (0 ≤ λ ≤ 1).^60^ Spinels constitute a vast family of compounds, which includes almost all of the main groups and transition metals. Due to their diverse compositions, electron/spin configurations, and valence states, spinels exhibit different magnetic, optical, electrical, and redox properties. HESs stand out from traditional spinels due to their distinctive characteristics in dynamics, structure, performance, and more. Similar to HEAs, the four key effects inherent in HESs primarily contribute to their distinction: high-entropy, lattice distortion, sluggish diffusion, and cocktail effects (Fig. 2).^61,62^

##### 2.2.2.1 High-entropy effect

The high-entropy effect is for the thermodynamic feasibility of the formation of solid solution phases. The phase stability in a multicomponent system is generally described by the Gibbs free energy, and its equation is as follows:

4 Δ*G*_mix_ = Δ*H*_mix_ − *T*Δ*S*_mix_

where Δ*G*_mix_, Δ*H*_mix_, and Δ*S*_mix_ are the changes in the Gibbs free energy, mixing enthalpy, and mixing entropy, respectively, and *T* is the thermodynamic temperature. A negative Δ*G*_mix_ implies that the system is conducive to forming a single-phase solution with random distributions, whereas a positive Δ*G*_mix_ indicates a tendency towards thermodynamically driven phase separation. Eqn (1) and (2) demonstrate that an increase in the number of mixed elements leads to a higher Δ*S*_mix_, which will become the decisive factor for the phase stability of the multicomponent system at sufficiently high temperatures.^63^

##### 2.2.2.2 Lattice distortion effect

The lattice distortion effect arises from variations in atomic sizes and unsymmetrical bonding among the constituent elements. The disparity in atomic sizes creates a state of thermodynamic nonequilibrium, which may theoretically lower the energy barrier for the adsorption, activation, and transformation of molecules involved in catalysis.^64^ This effect induces severe lattice strain in HESs, which impedes the dislocation movement and thus hinders the deformation of the crystal lattice under external forces. Tensile lattice strain can also cause an upwards shift of the d-band centre, resulting in stronger adsorption of reactant species on HES surfaces in catalytic reactions.^65,66^

##### 2.2.2.3 Sluggish diffusion effect

The sluggish diffusion effect is generated by variable diffusion rates, changes in chemical bonds, and divergence of potential energy, which is closely associated with the lattice distortion effect. A severe lattice distortion in HESs is expected to increase the diffusion activation energy of atoms within the lattice, thereby resulting in inhibited atomic diffusion, phase transformation and grain growth.^64^ The sluggish diffusion effect contributes to the formation of nanoscale HESs, allowing the HESs to maintain structural robustness even under extreme conditions.^53^

##### 2.2.2.4 Cocktail effect

The cocktail effect signifies a multifaceted synergistic impact resulting from the interaction among various element components in HESs. Many studies indicate that the performance of HESs is not merely a straightforward combination of the properties of each element but is instead unpredictable.^65^ The cocktail effect is affected not just by the average characteristics of constituent elements, but also by the other three effects discussed above.^64^ This effect is extensively reflected in various properties of HESs, encompassing their mechanical strength, corrosion resistance, and oxidation resistance.

In summary, HESs exhibit unique features arising from their complex composition and structure. The definition of HESs builds upon the principles of HEAs, incorporating both compositional and entropy-based criteria. In HESs, multiple cations and anions contribute to the increased configurational entropy, which plays a critical role in their stability and catalytic properties.^57^ The structural features of HESs, including the normal and inverse spinel arrangements, make them highly versatile in tuning their functional properties. The four core effects, including the high-entropy effect in thermodynamics, lattice distortion effect in structures, sluggish diffusion effect in dynamics, and cocktail effect in performance, collectively distinguish HESs from traditional spinels, making them promising candidates for advanced catalytic applications.^62^ For example, in electrocatalysis, the high-entropy and sluggish diffusion effects enhance the structural robustness of HESs, while lattice distortion optimizes the coordination environment of surface atoms and the adsorption energy of reactants and intermediates.^66^

### 2.3 Synthesis

The formation of HESs requires synthesis techniques capable of incorporating more than four elements into a single-phase spinel structure, ensuring homogeneity and phase stability. In addition, synthesized HESs with a high specific surface area, abundant active sites, and robust structural stability are more favorable in catalytic reactions. Therefore, a variety of methods have been used to fabricate HES materials for different catalytic purposes, including conventional synthesis methods (*i.e.*, solid-state and wet-chemical methods) and several emerging methods (*e.g.*, carbothermal shock synthesis).

#### 2.3.1 Solid-state methods

**Fig. 3:**
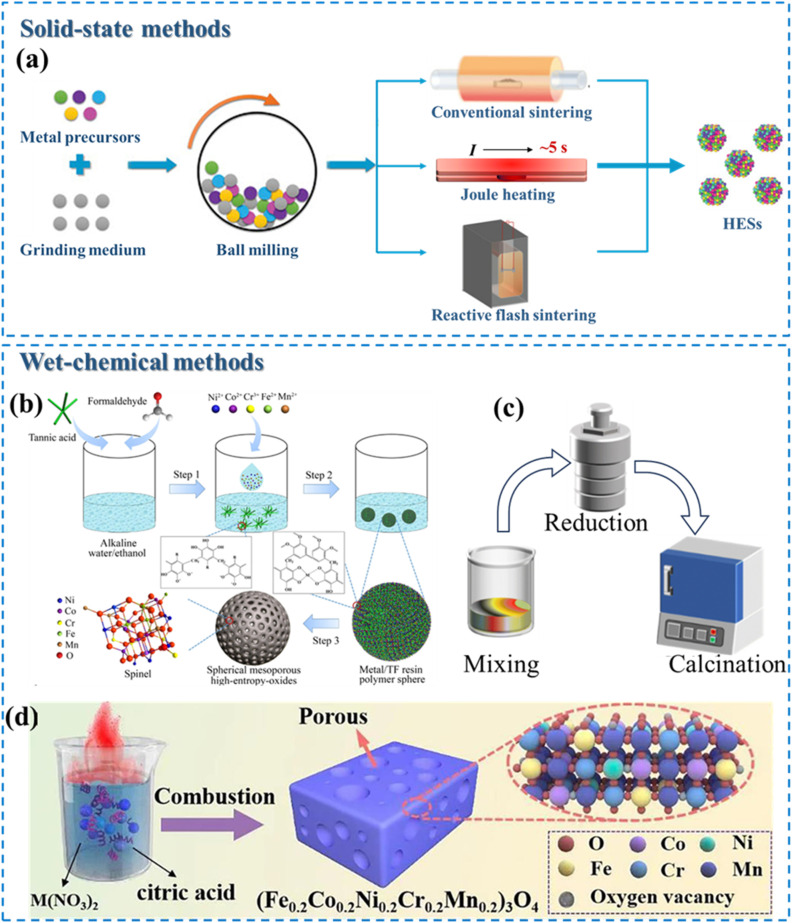
Conventional methods for HES synthesis. (a) Synthetic procedures of different solid-state methods. Reproduced with permission.^41^ Copyright 2022, John Wiley and Sons. Wet-chemical methods: (b) sol–gel method. Reproduced with permission.^67^ Copyright 2020, American Chemical Society. (c) Hydro-/solvothermal methods. (d) Solution combustion synthesis. Reproduced with permission.^68^ Copyright 2023, Elsevier.

Solid-state synthesis is the most conventional and widely used method for preparing HESs. This technique involves a direct reaction of precursor powders (*e.g.*, metal oxides, carbonates, or nitrates), which are first mixed by mechanochemistry and then subjected to high-temperature calcination (typically between 800 and 1200 °C), as shown in Fig. 3a. High-energy ball milling is an efficient tool for achieving a thorough mixing of metal precursors and producing a fine-grained powder. The localized heat produced by the combined mechanical energy and friction facilitates rapid atomic diffusion, which is particularly advantageous for synthesizing entropy-stabilized HESs.^34,43,69,70^ After the mechanical grinding, the resultant powders are sintered at high temperatures to initiate the nucleation of HES crystals. Conventional sintering relies on high temperatures and prolonged heating and cooling times, which could cause particle aggregation, secondary phase formation, and impurities. To overcome these drawbacks, rapid sintering techniques such as Joule heating, reactive flash sintering and spark plasma sintering have been emerging as promising alternatives. Ren *et al.* synthesized a spinel (Mg_0.2_Co_0.2_Ni_0.2_Cu_0.2_Zn_0.2_)Fe_2_O_4_ HEO by using ball milling followed by an ultrafast high-temperature sintering (UHS) technology in 5 seconds.^71^ This method enhances the preparation efficiency by four orders of magnitude compared to the conventional sintering method. Recently, Tyagi *et al.* developed a flash-sintering method to achieve higher densification with phase purity of (Mg_0.2_Ti_0.2_Zn_0.2_Cu_0.2_Fe_0.2_)_3_O_4_.^72^ Flash-sintered specimens exhibited greater hardness compared to conventionally sintered ones of the same density, which improved the cyclability of HESs in anode applications.

Solid-state methods are straightforward and can be easily scaled up for industrial production, making them suitable for large-scale applications. However, achieving a uniform distribution of multiple elements can be challenging due to limited diffusion rates under solid-state conditions.

#### 2.3.2 Wet-chemical methods

Compared with solid-state methods, wet-chemical methods such as the sol–gel technique, hydro-/solvothermal treatment, and solution combustion offer more control over the size, shape, and compositional distribution of HESs.^44,67,73,74^ In general, metal salts and various chemical reagents are mixed to produce a homogeneous precursor or precipitate. Then the mixture is calcined to remove the reagents to form single-phase HESs.

##### 2.3.2.1 Sol–gel method

This method has been commonly used for preparing nanostructured HESs with high surface areas, which are beneficial for catalytic applications. This method offers precise control over the chemical composition and homogeneity at the molecular level, leading to high-purity and uniformly mixed HESs. A typical sol–gel process involves the hydrolysis and condensation of metal alkoxides or salts to form a gel mixture, followed by drying and calcination to produce HESs. In addition, the sol–gel method allows for the incorporation of dopants or additives to tailor the morphology and structure of HES products. For example, Wang *et al.* reported the synthesis of spherical mesoporous HESs (Ni–Co–Cr–Fe–Mn oxide) by a sol–gel strategy using a plant polyphenol, formaldehyde, and metal salts as precursors (Fig. 3b).^67^ The HESs exhibited a high specific surface area (42–143 m^2^ g^−1^), large pore size (5.5–8.3 nm), and a unique spherical morphology (∼55 nm).

##### 2.3.2.2 Hydro-/solvothermal methods

This synthesis is a simple, energy-efficient, and well-controlled approach to producing HES nanomaterials.^44,50^ During the hydro-/solvothermal synthesis process, metal salts are mixed in water or an appropriate solvent and reduced at high temperatures and pressures in a sealed autoclave (Fig. 3c). By adjusting reaction parameters such as temperature, pressure, time, pH value, and precursor and additive concentrations, these methods enable the manipulation of material properties, including crystallinity, morphology, and size.^75^ Xiao *et al.* successfully synthesized a defect-rich (CoCrFeMnNi)_3_O_4_ HEO catalyst with a single-phase spinel structure *via* a hydrothermal method.^76^ Owing to the unique spinel structure and abundant oxygen vacancies, the catalyst displayed a high discharge capacity, superior rate capability, and long-term stability for vanadium redox flow batteries.

##### 2.3.2.3 Solution combustion synthesis (SCS)

The SCS is a rapid and self-sustained process to produce HESs.^17,77–79^ It involves dissolving metal nitrates (acting as oxidizers) and organic fuels (*e.g.*, urea, glycine, or citric acid) in a suitable solvent to form a homogeneous solution. Upon heating, an exothermic redox reaction occurs, generating intense heat and large amounts of gaseous byproducts, resulting in the formation of porous and finely dispersed solid products. He *et al.* first prepared nano-porous (Fe_0.2_Co_0.2_Ni_0.2_Cr_0.2_Mn_0.2_)_3_O_4_ with high grain boundary density by a low-temperature SCS method using metal nitrate as an oxidant and citric acid as a complexing agent (Fig. 3d).^68^ Due to the nano-porous structure and abundant grain boundaries, this catalyst exhibited excellent OER performance.

#### 2.3.3 Emerging methods

Many existing synthesis methods for HES production face challenges such as a low yield and high-energy input. A variety of innovative approaches are emerging to shorten the synthesis procedure, reduce production and energy costs, and prepare HESs with specific morphologies and structures for enhanced catalytic performance.

**Fig. 4:**
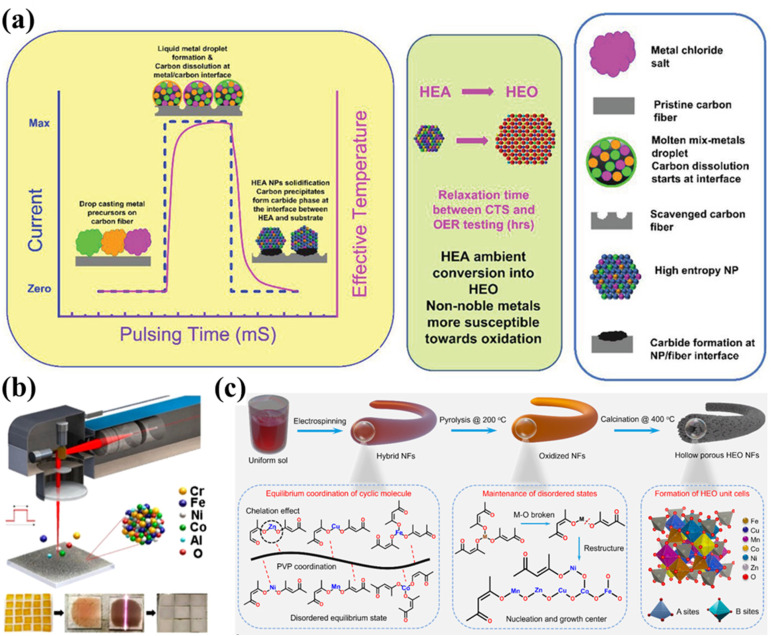
Emerging methods for HES synthesis. (a) Schematic illustration of the carbothermal shock (CTS) synthesis process within mS pulse intervals.^80^ (b) Laser-based shock methods. Reproduced with permission.^81^ Copyright 2021, American Chemical Society. (c) Electrospinning. Reproduced with permission.^82^ Copyright 2024, American Chemical Society.

Carbothermal shock (CTS) synthesis has already shown great promise in HEAs since 2018, offering unique advantages in terms of rapid production, controlled composition, and fine-tuning of properties.^83^ In recent years, this technique has also been used to synthesize HESs. It involves the rapid heating and cooling of a mixture of metal salt precursors and a carbon source, typically achieved within milliseconds to seconds at extremely high temperatures (1000–1700 °C).^80,84^ At elevated temperatures, metal salts initially undergo thermal reduction and then turn into liquid metal droplets, resting on a carbon-rich substrate. After rapid cooling to room temperature, the mixture forms a homogeneous HEA, which subsequently oxidizes to spinel oxides under an ambient atmosphere (Fig. 4a). This short reaction time is advantageous as it prevents excessive grain growth, thereby producing nanoscale materials. The resulting HESs are supported on carbon materials, which provide additional benefits such as enhanced electrical conductivity, mechanical strength, and surface area. The carbon support can also play a role in stabilizing the high-entropy spinel structure by preventing agglomeration and facilitating the dispersion of nanoparticles. These features are particularly beneficial for catalytic applications.

Abdelhafiz *et al.* reported that ternary to senary (FeNiCoCrMnV) HES nanoparticles on carbon fibers synthesized by CTS (rapid Joule heating and quenching) showed higher activity towards catalyzing the OER compared to an IrO_2_ catalyst with two orders of magnitude higher stability than IrO_2_, due to the stronger interaction with the substrate by metal–carbide bond activated during the OER process.^80^ In addition to thermal shock, laser shock has also been used to synthesize HESs. For example, Zhu *et al.* used a scaled-up (up to 51% mass ratio) laser-based shock method to fabricate HES nanoparticles on the surface of conductive carbon black at high speed.^85^ The carbon black substrate not only served as the conductive network within the HESs as an electrode material in LIBs but also provided optimal conditions for rapid heating and cooling processes used in nanoparticle synthesis. Yang *et al.* successfully used a pulsed laser to manufacture highly efficient electrocatalytic CoCrFeNiAl HES electrodes for the OER on various substrates (*e.g.*, indium tin oxide, Ni foam, and carbon cloth), as shown in Fig. 4b.^81^ This pulsed laser enables rapid synthesis with precise control over reaction conditions by adjusting parameters such as pulse energy, pulse duration, and scanning velocity.^81,86^

Similar to “shock”-based methods, several other techniques have been developed to synthesize HESs, including electrospinning, microwave-assisted synthesis, and spray pyrolysis. Electrospinning can produce nanofibers with high surface areas (Fig. 4c);^82,87–89^ microwave-assisted synthesis provides fast and uniform heating, leading to shorter reaction times and improved homogeneity of the final products.^24,90,91^ Spray pyrolysis, on the other hand, is highly versatile and scalable, making it suitable for large-scale production of finely dispersed HES powders.^92–94^

The structure and functionality of HESs are closely tied to the synthesis method employed, with significant variations observed at the microstructural level.^95^ Solid-state synthesis, while straightforward and scalable, often struggles with achieving uniform elemental distribution and reproducibility. Wet-chemical methods allow better control over the morphology, composition, and homogeneity, bringing HESs closer to the ideal definition and making them suitable for studies aimed at understanding the fundamental properties of HESs.^96^ Emerging “shock”-based synthesis techniques offer more efficient, scalable, and tailored production of HESs for enhanced catalytic performance, though they require specialized equipment and conditions. Continuous development and optimization of these synthesis techniques are essential for advancing high-entropy materials and unlocking their full potential in catalysis and other applications.

### 2.4 Characterization

The characterization of HESs mainly focuses on confirming their single-phase spinel structure and uniform distribution of elements. A series of techniques, including diffraction, microscopy, and spectroscopy, have been employed to characterize the local lattice structure and atomic distribution.^43,70,97–99^ More importantly, accurate characterization is crucial to identify and quantify active sites, investigate the effect of local structures (*e.g.*, oxygen vacancies and strain) on the catalytic performance, and finally reveal the underlying catalytic mechanisms.

**Fig. 5:**
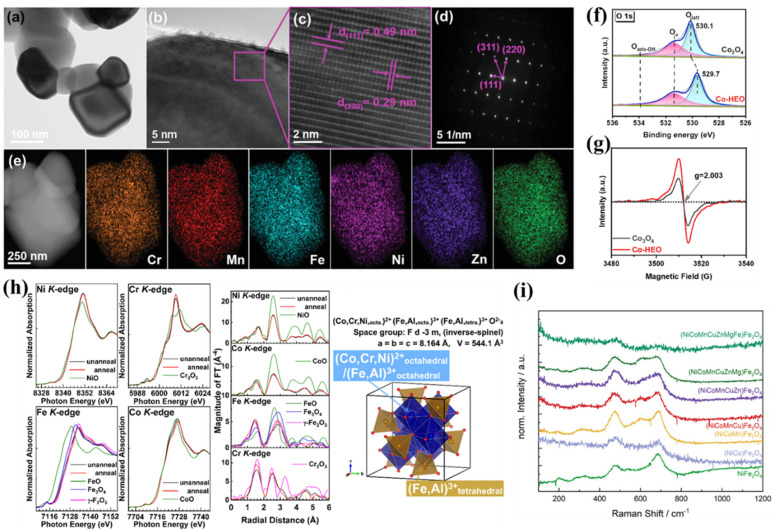
Characterization techniques of HESs. (a) TEM image, (b and c) HR-TEM images, (d) SAED pattern, and (e) STEM-EDS mapping of (CrMnFeNiZn)_3_O_4_. Reproduced with permission.^101^ Copyright 2023, John Wiley and Sons. (f) O 1s XPS spectra and (g) EPR spectra of Co_3_O_4_ and (CuMgNiZn)_1_Co_2_O_4_ for oxygen vacancy detection. Reproduced with permission.^51^ Copyright 2024, American Chemical Society. (h) Ni K-, Co K-, Fe K-, and Cr K-edge XANES and EXAFS spectra of NiCoFeCrAlO powder and schematic illustration of the inverse-spinel structure in this HES. Reproduced with permission.^81^ Copyright 2021, American Chemical Society. (i) Raman spectra of ferrites with increasingly more cations.^24^

X-ray diffraction (XRD) is a powerful tool to determine the crystal structure, phase purity, and lattice parameters of HES materials.^43,73^ High-resolution electron microscopy techniques, such as scanning electron microscopy (SEM) and transmission electron microscopy (TEM), are standard imaging methods. SEM can visualize the surface morphology, particle size, and elemental distribution of HESs. TEM especially high-resolution TEM (HR-TEM) can reveal the internal structure, crystallography, and defects within HESs and is crucial for studying the nanoscale features of HESs, including grain boundaries, dislocations, and the distribution of different elements within the spinel lattice.^21,100^ More precisely, high-angle annular dark-field scanning transmission electron microscopy (HAADF-STEM) can achieve atomic-scale resolution, enabling the visualization of individual atoms and their arrangements within the spinel structure. These methods are often combined with energy-dispersive spectroscopy (EDS or EDX) or electron energy loss spectroscopy (EELS) for elemental mapping. As shown in Fig. 5a–e, the nanoparticle morphology of a HES ((CrMnFeNiZn)_3_O_4_) was confirmed using the TEM image (Fig. 5a); lattice spacings of 0.49 and 0.29 nm shown by HR-TEM images (Fig. 5b and c) corresponded to the (111) and (220) planes of the cubic spinel oxide, respectively; the selected area electron diffraction (SAED) pattern (Fig. 5d) was well-indexed to a single-phase spinel structure; the even distribution of Cr, Mn, Fe, Ni, Zn, and O elements was verified by STEM-EDS elemental mapping (Fig. 5e).^101^

In contrast to EDS and EELS, which only examine a small sample area, spectroscopic techniques investigate the bulk and surface structures along with their associated chemical states. X-ray photoelectron spectroscopy (XPS) is frequently used to study the surface composition and chemical states of HESs, which are critical for understanding their catalytic behaviour since only surface atoms directly participate in heterogeneous reactions.^16,102^ Most studies on HESs also used XPS to identify the oxygen vacancies (Fig. 5f),^51^ but it is not reliable to take XPS as the only evidence for oxygen vacancy formation since many species hold similar binding energy.^103^ Electron paramagnetic resonance spectroscopy (EPR) has been a supplementary technique to precisely detect the oxygen vacancies in HESs (Fig. 5g).^51^ X-ray absorption spectroscopy (XAS) techniques, including extended X-ray absorption spectroscopy fine structure (EXAFS) and X-ray absorption near-edge structure (XANES), are valuable for further understanding chemical states of the constituent elements and their distribution over the octahedral (O_h_) and tetrahedral (T_d_) co-ordination environment in HESs.^97,104^ XANES examines the oxidation states and coordination chemistry of the tested elements, while EXAFS provides detailed information on the distances between the adsorbing atom and its neighboring atoms, the coordination number, and the types of neighboring atoms. Fig. 5h shows the Ni K-, Co K-, Fe K-, and Cr K-edge XANES and EXAFS spectra of a HES (NiCoFeCrAlO) and a modelled inverse-spinel structure of this HES based on the results.^81^

Other techniques such as Raman spectroscopy, Fourier-transform infrared (FTIR) spectroscopy, and Mössbauer spectroscopy are also used to characterize HESs.^24,99,105^ These methods provide detailed insights into the vibrational properties, chemical bonding, surface chemistry, oxidation states, and magnetic properties, complementing other analytical techniques for a comprehensive understanding of these complex materials. For example, as shown in Fig. 5i, Raman spectra of ferrites with increasingly more cations nearly all exhibited a band between 450 and 500 cm^−1^, which could correspond to the T_2g_ bands arising from asymmetric stretching and bending M–O vibrations at octahedral sites in an inverted binary spinel. In addition, the bands became significantly broader as the number of cations increased, indicating the increased cation disorder also on the octahedral sites.^24^

Conventionally, the structure of HESs is characterized and compared before and after a reaction *via ex situ* characterization techniques described above to identify structural changes, surface modifications, and the nature of active sites, thus gaining insights into the catalytic mechanisms.^44,45,106^ However, in many cases, the structure of a catalyst often differs during the catalytic reaction compared to its structure before or after the reaction, which may lead to a misunderstanding of mechanisms.^107^ In addition, the process of preparing, handling, and transferring samples for *ex situ* measurements possibly causes changes in the reacted samples (*e.g.*, contamination and relaxation). *In situ*/*operando* characterization techniques enable the real-time monitoring and characterization of the local structure/property/performance, providing a deeper understanding of reaction processes and mechanisms. Many *in situ*/*operando* characterization techniques, including XRD, XPS, XAS, Raman, and DRIFTS (diffuse reflectance infrared Fourier transform spectroscopy), have been applied for HES characterization to capture the average atomic or molecular information under real-working conditions.^75,108–112^ For example, Chang *et al.* used *operando* quick-scanning XAS to investigate the lithiation/delithiation mechanism of the (CrMnFeNiCu)_3_O_4_ HES.^113^ The regime was revealed *via* examining valence/coordination state variations, transition steps, redox sequences, reversibility, and redox overpotential of multiple electroactive centers in the (CrMnFeNiCu)_3_O_4_ electrode. Wu *et al.* applied *in situ* TEM to monitor the microstructural evolution of (Cr, Mn, Fe, Co, and Ni)_3_O_4_ during calcination, offering valuable insights for optimizing HEO synthesis *via* solid-state methods.^114^

To assess the properties of HESs, various characterization methods are required to examine their elemental compositions, crystallographic features, microstructure and morphologies, and chemical states. Techniques such as XRD, SEM, TEM, XPS, XAS, EPR, Raman, and FTIR provide detailed insights into the structural and compositional information of HESs, as well as identifying active sites and elucidating catalytic mechanisms. While conventional *ex situ* techniques are crucial for analyzing structural changes, *in situ*/*operando* methods offer real-time insights into catalytic reactions under working conditions. Additionally, advanced characterization techniques, such as three-dimensional atomic electron tomography (3D-AET) and four-dimensional STEM (4D-STEM), are also being explored within the high-entropy community to achieve atomic-level insights into HEMs.^115,116^ The combination of these characterization methods allows for a comprehensive understanding of structure–property relationships of HESs, which is critical for on-demand HES catalyst design and advancing their catalytic applications.

## 3. Catalytic applications

As discussed in Section 2.2.2, the marriage of high-entropy design and spinel structure makes HESs exhibit remarkable physicochemical properties with great potential and special advantages in catalytic applications. This section summarizes the recent advances in HESs in thermocatalysis, electrocatalysis, and photocatalysis. Most importantly, the structure–property–performance relationships of HESs and catalytic reaction mechanisms are highlighted.

### 3.1 Thermocatalysis

In the realm of thermocatalysis, HESs have shown promising performance in reactions such as hydrocarbon reforming, oxidation, and hydrogenation reactions. Their high thermal stability and multiple active sites allow them to withstand harsh reaction conditions and provide enhanced catalytic performance.

#### 3.1.1 Hydrocarbon reforming reactions

**Fig. 6:**
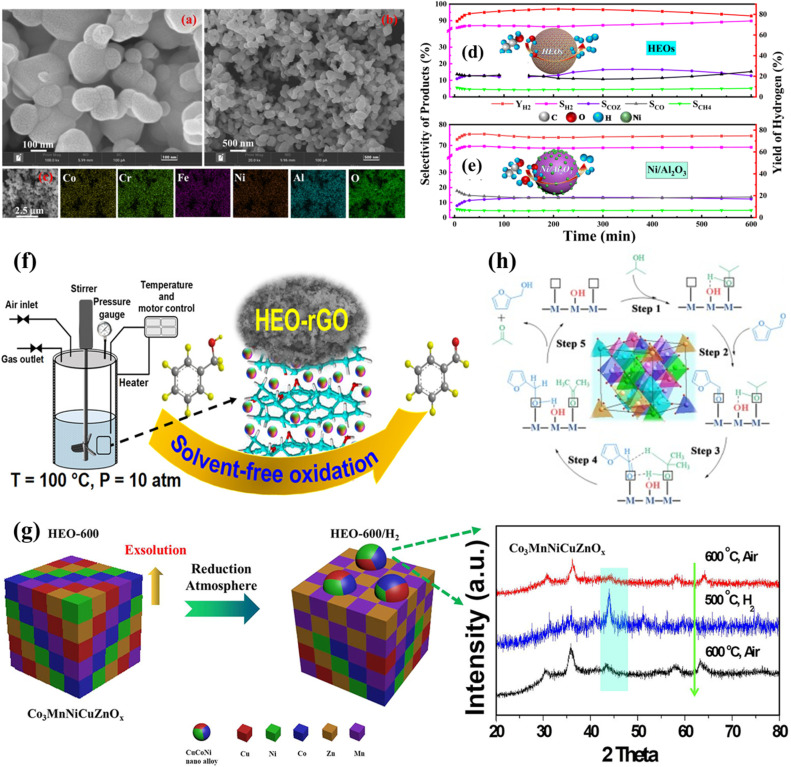
HESs for thermocatalysis. (a and b) The SEM images of the HES (CoCrFeNiAl)_3_O_4_, (c) the EDS mapping of the HESs, and the product selectivity and H_2_ yield of (d) HESs and (e) Ni/Al_2_O_3_ after 10 h of ethanol steam reforming.^50^ (f) (CoFeMnCuNiCr)_3_O_4_ on reduced graphene oxide (rGO) for solvent-free aerobic oxidation of benzyl alcohol. Reproduced with permission.^105^ Copyright 2024, American Chemical Society. (g) Schematic illustration of *in situ* exsolution of CuCoNi nanoalloys from HES (Co_3_MnNiCuZnO_*x*_) during CO_2_ hydrogenation, with the XRD patterns of HES, HES/H_2_, and the re-oxidized sample. Reproduced with permission.^48^ Copyright 2021, American Chemical Society. (h) Proposed mechanism for the transfer hydrogenation (CTH) reaction of furfural with 2-PrOH on holey lamellar HES nanocrystals with frustrated Lewis pairs. Reproduced with permission.^118^ Copyright 2023, Elsevier.

These reactions involve the conversion of hydrocarbons (typically alkanes) into H_2_ and other valuable products, such as CO or syngas, which play an important role in renewable energy technologies. HES catalysts have demonstrated satisfactory high-temperature catalytic activity, selectivity and stability (improved anti-coking and anti-sintering ability) during these processes.^117^ Wang *et al.* first reported an application of a HES catalyst ((CoCrFeNiAl)_3_O_4_) in ethanol steam reforming (ESR) for H_2_ production. The HESs were composed of uniformly dispersed nanoparticles with five metal species, observed by SEM and EDS mapping (Fig. 6a–c).^50^ They achieved 81% of H_2_ yield with 85% selectivity at 600 °C, higher than those of Ni/Al_2_O_3_ (Fig. 6d and e), and exhibited high thermal stability, with a consistently high level of H_2_ yield and selectivity in 55 h. XPS and EPR identified abundant oxygen vacancies formed in the HES catalyst, which were further enriched in a H_2_ atmosphere as the M–O bond opened, thus promoting the catalytic activity during ESR. The increase in oxygen vacancies significantly enhances the oxygen mobility of HESs compared to traditional oxide supports, thereby providing more active sites.^119^ The characterization results of the spent catalysts indicated that the HESs had an ability to self-reorganize, making the metals spill out of the HES bulk phase as active species for H_2_ production and randomly dissolving back into the parent metal oxide cells after the reaction instead of agglomerating on the catalyst surface.

In a recent study, a HES ferrite (Co_0.2_Ni_0.2_Cu_0.2_Zn_0.2_Mg_0.2_)Fe_2_O_4_ catalyst was prepared for H_2_ production *via* steam reforming of co-pyrolysis volatiles of polypropylene and waste cooking oil.^120^ The HES catalyst produced 58.43 mmol g^−1^ of H_2_ with a composition of 65.17% at 750 °C, 22.3 times higher than that of 10%Fe/Al_2_O_3_ and 10%Ni/Al_2_O_3_, and exhibited an extended service life of 9 h without compromising H_2_ selectivity with a H_2_ yield of 510.2 mmol g_cat._^−1^ s^−1^. The improved reductivity originated from the strong C–C breaking ability of reactive oxygen species caused by oxygen vacancies (OVs) and Fe species and the synergistic C–H bond cleavage by Fe, Ni, Co, Cu, and Zn species. Specifically, the elevated temperature caused the reduction of metals, mainly Fe^3+^ to Fe^2+^ and Fe^2+^ to Fe^0^. To ensure charge balance during the transition of Fe species, a larger number of OVs were formed on the surface, which can activate gaseous oxygen and produce unstable oxygen species to facilitate catalytic reactions. Compared to single-metal-loaded Fe and Ni catalysts, the HESs exhibited better catalytic effects, indicating the catalytic synergy among different metals.

HESs have also been used in the chemical looping process for efficient and stable syngas and H_2_ coproduction. Zhong *et al.* utilized (Ni_0.2_Co_0.2_Ca_0.2_Cu_0.2_Mg_0.2_)Fe_2_O_4_ in chemical looping reforming coupled with water splitting (CLR-WS) for highly efficient and stable hydrogen production using CH_4_ as a fuel.^121^ Among the spinel catalysts with different configuration entropies, only HESs exhibited a high H_2_ yield of 12.66 mmol per gram of oxygen carriers with a purity of 99.1% at 700 °C and maintained 94.76% of their theoretical oxygen transfer capacity (OTC) during 100 cycles, further validating the benefits of multi-element synergy in HESs.

#### 3.1.2 Oxidation reactions

Oxidation of various substrates, including alcohols, hydrocarbons, CO, and others, constitutes a significant category of reactions in thermocatalysis, for which the development of effective catalysts at high temperatures is highly desired. Recent studies demonstrated that HESs could provide an outstanding platform for these reactions, improving product yields and reducing by-products. Mehrabi-Kalajahi *et al.* immobilized (CoFeMnCuNiCr)_3_O_4_ nanoparticles on reduced graphene oxide (rGO) to produce a HEO–rGO nanocomposite for solvent-free aerobic oxidation of benzyl alcohol (Fig. 6f) and achieved up to 10.36% conversion and 78.5% selectivity of benzaldehyde in 4 h.^105^ Abundant redox couples Co^2+^/Co^3+^, Fe^2+^/Fe^3+^, Mn^2+^/Mn^3+^, Cu^+^/Cu^2+^, Ni^2+^/Ni^3+^, and Cr^2+^/Cr^3+^ and oxygen vacancies were presented in the catalyst, which can adsorb oxygen species and accelerate the oxidation of benzyl alcohol. The rGO structure provided a large surface area favorable for efficient adsorption and selective oxidation of benzyl alcohol. Thus, the synergistic effects of the metal cations and oxygen vacancies in the HEO and the structure of rGO led to the excellent performance of HEO–rGO nanocomposites. Yuan *et al.* prepared HES (FeCoNiCrMn)_3_O_4_*via* two different methods to identify the role of oxygen vacancies in HESs for the selective C–H oxidation of *p*-chlorotoluene to *p*-chlorobenzaldehyde.^122^ Compared to the HES by physically mixing, the one prepared by coprecipitation was found to have more oxygen vacancies verified by H_2_ temperature-programmed reduction (H_2_-TPR), O_2_ temperature-programmed desorption (O_2_-TPD) and O 1s XPS. Density functional theory (DFT) calculations revealed that the presence of oxygen vacancies in the HES was favorable for the adsorption of *p*-chlorotoluene toward the formation of a critical *ClPhCH_2_O intermediate and the desired *p*-chlorobenzaldehyde.

In addition, HESs have also been explored to catalyze CO oxidation,^123^ CH_4_ oxidation,^124^ propane oxidation,^125^ soot combustion,^126^*etc.*, highlighting the important role of multicomponents, oxygen vacancies and entropy stabilization in HESs for high-temperature oxidation reactions.

#### 3.1.3 Hydrogenation reactions

Many hydrogenation reactions over metal catalysts are performed at high temperatures, making HESs ideal candidates because of their entropy-stabilized structures which exhibit excellent resistance to sintering. CO_2_ reduction is a crucial process for mitigating climate change by converting CO_2_ into valuable chemicals and fuels. Zhao *et al.* synthesized a HES Co_3_MnNiCuZnO_*x*_ catalyst with a single inverse spinel structure and performed a reverse water–gas shift reaction (RWGS) to convert CO_2_ into CO.^48^ The presence of abundant oxygen vacancies and exposed active sites (CuCoNi nanoalloys and metal–oxide interfaces in Fig. 6g) in a reducing atmosphere facilitated H_2_ dissociation and CO_2_ activation, which resulted in high catalytic activity (48% CO_2_ conversion and 94% CO selectivity) at 500 °C, outperforming binary and ternary metal oxide catalysts. The highly dispersed CuCoNi nanoparticles on the HES matrix during the RWGS process guaranteed long-term thermal stability (>100 h) and good resistance to sintering. The synergy of five metals in the HES was also confirmed in this study because increasing the amount of any one of the five elements did not significantly change the catalytic activity. However, in Ayano's study, Mn, Fe, Co, and Ni within (CoCrFeMnNi)_3_O_4_ were identified as the active sites in the RWGS reaction, while Cr played a crucial role in stabilizing the spinel structure; this conclusion was drawn by comparing the activities of the HES (CoCrFeMnNi)_3_O_4_ and five quaternary spinel oxides (each missing element) characterizing the spent catalysts.^127^ Any compositional adjustment will lead to changes in their overall structures and properties of HESs, making it challenging to directly compare findings across different studies. In addition to exhibiting excellent high-temperature performance, (Ni_0.2_Mg_0.2_Cu_0.2_Zn_0.2_Co_0.2_)Fe_2_O_4_ HES also demonstrated SO_2_-tolerant capacity.^112^*In situ* DRIFT, SO_2_-TPD, *in situ* S 2p XPS and the DFT results confirmed that the low SO_2_ adsorption energy of (Ni_0.2_Mg_0.2_Cu_0.2_Zn_0.2_Co_0.2_)Fe_2_O_4_ was attributed to the lower Gibbs free energy.

Ma *et al.* synthesized HES nanocrystals (CrMnFeCoNiO) with abundant frustrated Lewis pairs (FLPs) for hydrogenation of unsaturated compounds, displaying superior catalytic activity and cycling performance under mild conditions.^118^ The FLPs on the HESs were composed of oxygen vacancies as Lewis acid sites and proximal surface hydroxyls or surface lattice oxygen as Lewis base sites. Attenuated total reflectance-infrared spectroscopy (ATR-IR) analysis and DFT calculations revealed that active regions between FLPs provide a stronger driving force for dissociating alcohols and activating the carbonyl groups of substrates, which enhanced catalytic activity (Fig. 6h).

### 3.2 Electrocatalysis

Electrocatalysis drives sustainable and clean energy conversion and storage technologies, including water electrolyzers, fuel cells, batteries, and supercapacitors. The diversity in composition and structures of HESs allows for the rational regulation of catalyst surface chemistry, enhancing their efficiency and selectivity in electrochemical transformations, especially in oxygen catalysis and Li-ion batteries.

#### 3.2.1 Oxygen evolution reaction

The oxygen evolution reaction (OER) plays a crucial role as an electrode reaction in water-splitting and metal–air batteries. The OER (2H_2_O → O_2_ + 4H^+^ + 4e^−^ in acidic media/4OH^−^ → 2H_2_O + O_2_ + 4e^−^ in alkaline media) requires the migration of four electrons with several intermediate reactions, resulting in sluggish kinetics. The activities of IrO_2_ and RuO_2_ for the OER are among the highest reported to date, but high cost and low stability limit their wide application.^128,129^ Thus, the bottleneck is to develop noble-metal-free OER electrocatalysts to achieve low overpotential, robust stability, and low cost. HESs have been widely studied as promising OER catalysts due to their intriguing properties.^16,20,111,130^

**Fig. 7:**
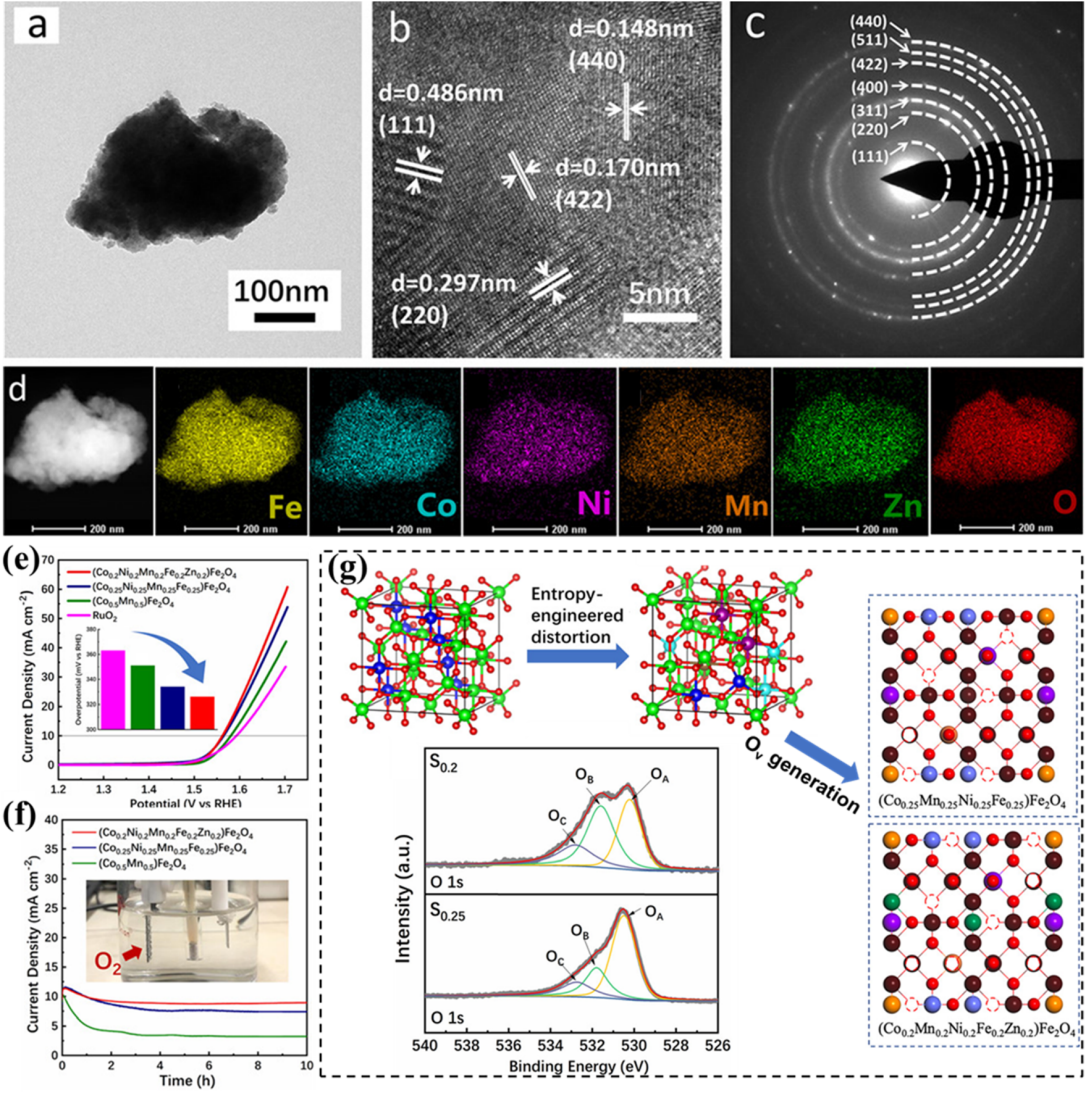
HESs for the OER. (a) TEM image, (b) HRTEM image, (c) SAED pattern, (d) HAADF-STEM image and elemental mapping of (Co_0.2_Mn_0.2_Ni_0.2_Fe_0.2_Zn_0.2_)Fe_2_O_4_, (e) LSV curves of different spinels and commercial RuO_2_ (the inset figure shows the overpotential at 10 mA cm^−2^), (f) chronoamperometry curves, and (g) schematic illustration of the formation of oxygen vacancies on the (400) surfaces of (Co_0.25_Mn_0.25_Ni_0.25_Fe_0.25_)Fe_2_O_4_ (S_0.25_) and (Co_0.2_Mn_0.2_Ni_0.2_Fe_0.2_Zn_0.2_)Fe_2_O_4_ (S_0.2_) caused by the lattice distortion in the O-neighbored octahedral and tetrahedral sublattices with their XRD patterns. Reproduced with permission.^131^ Copyright 2020, American Chemical Society.

Wang *et al.* reported the application of HESs ((Co, Cu, Fe, Mn, Ni)_3_O_4_) in the OER, which is also the first time that a HEO has served as an efficient catalyst in electrocatalysis. Using hydrophilic multi-walled carbon nanotubes (MWCNTs) to support HES nanoparticles, the resultant HES achieved an overpotential of 1.58 V at a current density of 10 mA cm^−2^ in 1 M KOH electrolyte and maintained its activity after 12 h of the stability test.^44^ The better electrocatalytic activity of HESs than that of mixed metal oxides with fewer elements is attributed to the diverse valence states and chemical components of the oxides/hydroxides in the HES layers, which provide numerous intermediates and thus can overcome the kinetic barriers more easily. *Operando* electrochemical impedance spectroscopy (EIS) analysis revealed that M^3+^ (M = Co^3+^, Fe^3+^, Mn^3+^ and Ni^3+^)_Oh_ in the octahedral site is responsible for surface electric double-layer capacitance, while the M^2+^ (M = Co^2+^, Fe^2+^, Mn^2+^ and Ni^2+^)_Td_ species in the tetrahedral site were responsible for water oxidation. Zhang *et al.* prepared entropy-stabilized (Co_0.2_Mn_0.2_Ni_0.2_Fe_0.2_Zn_0.2_)Fe_2_O_4_*via* high-energy ball milling with a single-phase spinel structure (Fig. 7a–d).^131^ The material exhibited both high activity (336 mV at 10 mA cm^−2^ in 1 M KOH) and stability (maintaining an 89.4% current density after 10 h) in the OER (Fig. 7e and f). XPS analysis revealed that the enhanced OER performance of HESs, compared to two lower-entropy spinels, was attributed to significant lattice distortion and increased configurational entropy resulting from the disordered occupation of multivalent cations. The distorted lattice promoted the formation of high-density oxygen vacancies on the HES surface (Fig. 7g), which can serve as the active sites for H_2_O absorption. Higher configurational entropy prevented rapid degradation of the lattice structure, which helped maintain oxygen vacancies and thus preserved the active sites during extended OER testing.

**Fig. 8:**
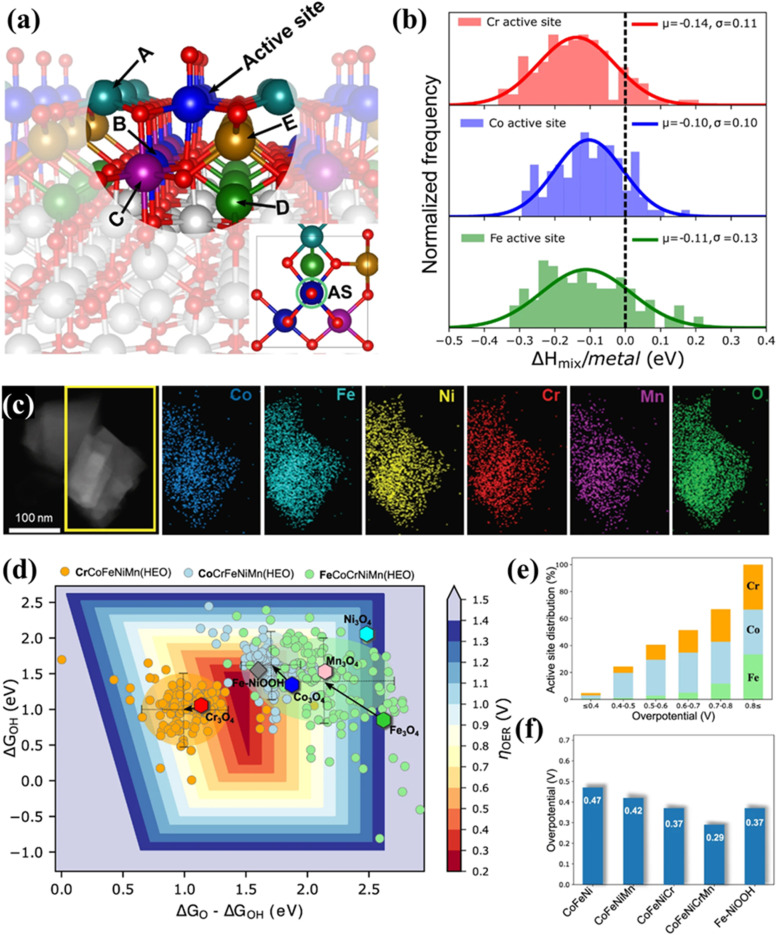
The combination of theoretical and experimental approaches to examine the OER activity and stability of HESs. (a) The HES impurity model containing the OER active site in the centre with adsorbed O* (red) surrounded by 5 metal neighbors (A to E for Cr, Co, Mn, Ni, and Fe) (the inset figure shows the top view of HES containing active sites (ASs)), (b) relative mixing enthalpy per mixing metals for different active sites on the HES surface as referred to their bulk systems, (c) EDS maps of synthesized HES (Co, Fe, Ni, Cr, and Mn), (d) OER activity volcano plot as a 2D heat map of overpotentials based on binding energy calculation of O* and OH*, and scaled OOH* values for HES systems, grouped by the active site along with the pure spinel systems, (e) active site (Cr, Co, and Fe) distribution as a function of OER overpotential for HEO systems and (f) predicted activity trends for different spinel oxide systems with explicitly calculated OOH*.^132^

Due to the structural complexity, the regime behind the enhanced performance of HESs still lacks solid experimental evidence and molecular-level understanding. Baek *et al.* combined theoretical and experimental approaches to examine the OER activity and stability of HESs including Co, Fe, Ni, Cr, and Mn.^132^ They computationally developed a feasible HES impurity model (Fig. 8a) and used it to calculate the mixing enthalpies (Fig. 8b), demonstrating the thermodynamic stability of the HES system. Guided by the theoretical studies, they synthesized a HES with homogeneous mixing of each element (Co, Fe, Ni, Cr, Mn, and O) shown in Fig. 8c, which exhibited superior OER activity (∼307 mV at 10 mA cm^−2^ in 1 M KOH) and durability (∼12% increment of the overpotential over 168 h), outperforming lower-entropy oxides. The intermediate (O* and OH*) binding energies for three different active sites (Cr, Co, and Fe) were further calculated based on this model, showing that the overall activity was primarily contributed by Co, followed by Cr and Fe as well as oxyhydroxide (Fig. 8d–f). The high OER activity in HESs originated from the wider intermediate adsorption energy distributions due to the local strain in the active metal site–oxygen bonds.

#### 3.2.2 Oxygen reduction reaction (ORR)

Like the OER, the complex and slow kinetics of the ORR have been the bottleneck in energy technologies, including fuel cells and metal–air batteries. It is widely recognized that the ORR can proceed through two distinct pathways: a two-electron (2e^−^) process that produces hydrogen peroxide (H_2_O_2_), or a four-electron (4e^−^) pathway that generates H_2_O. The 2e^−^ pathway is advantageous for the synthesis of valuable chemicals H_2_O_2_, whereas the 4e^−^ pathway is preferred for fuel cells and metal–air batteries due to its high energy conversion efficiency. Most current studies of HESs focus on the 4e^−^ ORR route, utilizing HESs as cathode materials for fuel cells or metal–air batteries.^23,133–136^

**Fig. 9:**
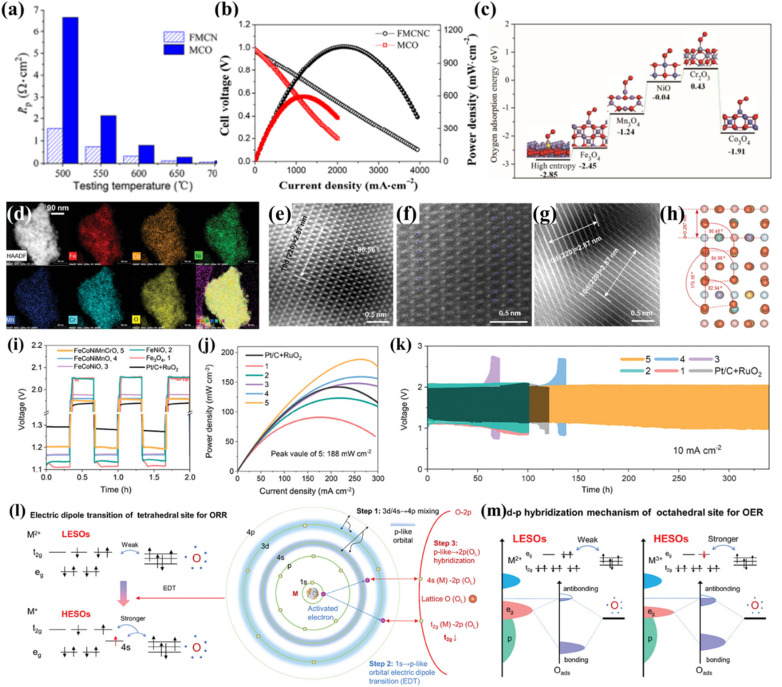
HESs for the ORR. (a) The polarization resistance (*R*_p_) values of the cell using Fe_0.6_Mn_0.6_Co_0.6_Ni_0.6_Cr_0.6_O_4_ (FMCNC) and Mn_1.6_Cu_1.4_O_4_ (MCO) cathodes tested at different temperatures, (b) the fuel cell performance, and (c) O_2_ adsorption energy on the surface of HES, Fe_3_O_4_, Mn_3_O_4_, Co_3_O_4_, NiO, and Cr_2_O_3_.^49^ (d) STEM image and EDS mapping, (e–g) HAADF-STEM images and (h) the corresponding simulated model of HESs (FeCoNiMnCrO), (i) charge–discharge curves of ZABs at initial three cycles at 10 mA cm^−2^ using different spinels and commercial catalysts (Pt/C and RuO_2_) electrodes, (j) powder density, (k) long-term cycling curves at 10 mA cm^−2^, (l) high-entropy induced robust electric dipole transition in tetrahedral sites for the ORR, and (m) high-entropy induced strong d–p hybridization of octahedral sites for the OER. Reproduced with permission.^137^ Copyright 2024, John Wiley and Sons.

For example, Xu *et al.* used Fe_0.6_Mn_0.6_Co_0.6_Ni_0.6_Cr_0.6_O_4_ for the first time as the cathode for proton-conducting solid oxide fuel cells (H-SOFCs), which possessed a much better ORR activity (a polarization resistance of 0.057 Ω cm^2^ at 700 °C shown in Fig. 9a) than a traditional Mn_1.6_Cu_1.4_O_4_ spinel cathode, benefiting the cathode performance (a peak power density of 1052 mW cm^−2^ shown in Fig. 9b).^49^ The first-principles calculation showed that the HES had lower O_2_ adsorption energy than individual oxides (Fe_3_O_4_, Mn_3_O_4_, Co_3_O_4_, NiO, and Cr_2_O_3_), indicating more thermodynamically favorable adsorption of O_2_ on HESs (Fig. 9c). Compared to Mn_1.6_Cu_1.4_O_4_, the HES cathode exhibited higher protonation ability and had a closer O p-band centre to the Fermi level, thereby enhancing the catalytic ORR activity of the cathode.

Recently, Zhang *et al.* demonstrated that a high-entropy strategy was able to activate and stabilize the tetrahedral sites (potential active sites for the ORR) and enhance the activity of octahedral sites (potential active sites for the OER) in spinel oxides, thereby effectively decoupling the ORR/OER in zinc–air batteries (ZABs).^137^ The designed HESs displayed the uniform distribution of Fe, Co, Ni, Mn, Cr, and O within the particles (Fig. 9d) and had significant surface reconstruction on the (220) exposure facet due to severe lattice-distortion effects (Fig. 9e–h). The HESs exhibited comparable catalytic activity but superior stability to Pt/C for the ORR as well as superior performance in ZABs (a narrow charge–discharge voltage gap of 0.74 V, a peak power density of 188 mW cm^−2^, and outstanding long-term cycling durability, as shown in Fig. 9i–k). Combining theoretical and experimental investigations, the superior electrocatalytic performance was attributed to the electron structure redistribution induced by the high-entropy effect. Specifically, the significant lattice distortion in the HESs triggered an intense 1s → 4s electric dipole transition and strong t_2g_(Co)/e_g_(Ni)–2p(O_L_) hybridization, resulting in low-valence Co tetrahedral sites (Co_th_) and high-valence Ni octahedral sites (Ni_oh_), as shown in Fig. 9l and m. This created dipolar dual-active sites capable of efficiently decoupling the OER/ORR. This study also first used unpaired t_2g_ occupancy as an electronic descriptor for ORR activity for tetrahedral sites and demonstrated that the entropy engineering effectively regulated the electrocatalytic activity descriptors (t_2g_ and e_g_) of the metal sites for the ORR and OER in HESs.

A HES ((Fe_0.2_Zn_0.2_Co_0.2_Ni_0.2_Cu_0.2_)Fe_2_O_4_) has also been evaluated as a 2e^−^ ORR electrocatalyst for H_2_O_2_ production and exhibited a high H_2_O_2_ selectivity ranging from 69.48% to 85.78% under a potential window of 0.2–0.65 V with durability up to 24 h, which were attributed to the high concentration of oxygen vacancies and the synergistic interaction of multiple components.^138^

#### 3.2.3 Li-ion batteries

HESs are emerging anode materials for Li-ion batteries (LIBs).^41,139^ Compared to rocksalt-type HEOs as a LIB anode, the spinel structure in HESs can offer three-dimensional Li^+^ diffusion channels.^140,141^ In addition, the abundant oxygen vacancies induced by multivalent cations at two distinct Wyckoff positions within the spinel structure can modulate the electronic structure and facilitate ionic conduction.^136,142^ Ever since the introduction of HESs as potential anode materials for LIBs in 2020, there has been a continuous surge of energy storage applications focusing on HES materials. Chen *et al.* first reported a novel HES (Mg_0.2_Ti_0.2_Zn_0.2_Cu_0.2_Fe_0.2_)_3_O_4_ incorporating heterovalent cations (Fe^3+^ and Ti^4+^) as anode materials for LIBs, which enriched the spinel-HEOs diversities. The as-obtained (Mg_0.2_Ti_0.2_Zn_0.2_Cu_0.2_Fe_0.2_)_3_O_4_ particles delivered a charge–discharge capacity of 504 mA h g^−1^ at a current density of 100 mA g^−1^ after 300 cycles and a rate capacity of 272 mA h g^−1^ at 2 A g^−1^.^34^ The high-entropy spinel structure not only buffered the volumetric changes during lithiation/delithiation processes, but also offered increased surface sites for lithium storage and promoted the charge/electrolyte reaction. Meanwhile, Nguyen *et al.* demonstrated that a non-equimolar HES (FeCoNiCrMn)_3_O_4_ exhibited a reversible capacity of 1235 mA h g^−1^ at 20 mA g^−1^, the highest among all the HEOs ever reported.^143^ The distributed metallic phases ensured the high electronic conductivity and abundant oxygen vacancies in HES, which facilitated Li^+^ diffusion and contributed to the high rate capacity of the electrode (500 mA h g^−1^ at 2 A g^−1^). Even without dummy MgO structural pillars, the HES still had much better electrode cycling stability, with 90% capacity retention after 200 cycles, compared to common conversion-type oxide anodes.

**Fig. 10:**
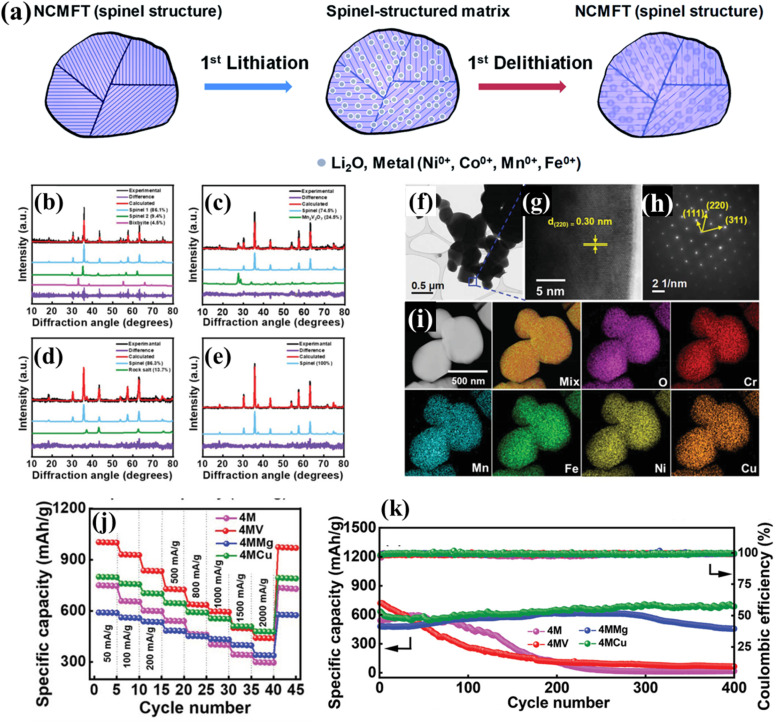
HESs for LIBs. (a) Charge storage mechanism during initial lithiation and delithiation processes.^109^ XRD patterns of (b) (CrNiMnFe)_3_O_4_(4M), (c) (CrNiMnFeV)_3_O_4_(4MV), (d) (CrNiMnFeMg)_3_O_4_(4MMg), and (e) (CrNiMnFeCu)_3_O_4_(4MCu); (f) TEM image, (g) high-resolution lattice image, (h) SAED pattern, and (i) HAADF image and EDS mapping of 4MCu; (j) comparative rate capability of various electrodes, and (k) cycling stability of various electrodes at 500 mA g^−1^ for 400 cycles. Reproduced with permission.^108^ Copyright 2022, John Wiley and Sons.

Chen *et al.* synthesized (Ni_0.2_Co_0.2_Mn_0.2_Fe_0.2_Ti_0.2_)_3_O_4_ as an anode material for LIBs, exhibiting a capacity of ∼560 mA h g^−1^ at 100 mA g^−1^ with an excellent capacity retention of 100% after 100 cycles.^109^ To investigate the lithium storage mechanisms, a series of characterization methods including *in operando* XANES, *in operando* XRD, *in operando* TXM (transition X-ray microscopy), *ex situ* XPS, and *ex situ* TEM were conducted to understand the redox reactions and structural changes of the HES during lithiation and delithiation. Combining these results, a mechanism model was proposed (Fig. 10a). During the lithiation process of the HES anode, Ni, Co, Mn, and Fe in the HES were all reduced to the metallic state, and Li ions formed Li_2_O by a reaction with the metal oxide, while Ti ions formed spinel LiTi_2_O_4_, which helped maintain the spinel structure and facilitated the reduced cations to re-occupy the original sites. During the delithiation process, most of the metal nanograins were oxidized back to their original spinel structure. As a result, no significant volume expansion of the HES particles during the lithiation and delithiation processes was observed due to the high-entropy stabilization of the lattice. Patra *et al.* prepared a series of Co-free HESs (V/Mg/Cu was added to quaternary medium-entropy (CrNiMnFe)_3_O_4_, 4M) for LIBs and demonstrated that chemical composition of high-entropy oxides was crucial for achieving phase purity and optimal charge–discharge performance.^108^ Among the three HESs, only 4MCu had a single-phase spinel structure verified by XRD (Fig. 10b–e) and HRTEM (Fig. 10f–i), which also exhibited the highest rate capacity and cyclability (480 mA h g^−1^ at 2 A g^−1^ and almost no capacity decay after 400 cycles as shown in Fig. 10j and k). The high phase purity can maximize the entropy stabilization effects, which helped maintain the crystalline framework of 4MCu during the lithiation/delithiation process and improve its electrode reversibility. In addition, the highest oxygen vacancy concentration of 4MCu among the three HESs was believed to be the decisive factor in its high capability and reversibility, which can modulate the electronic structure and promotes Li^+^ transport.

To compare different HESs for LIBs conveniently, Table 1 presents the element composition and electrochemical performance of the current HES anode materials. In addition, HESs have also been reported for the application of electrode materials in sodium ion batteries,^92^ lithium–sulfur batteries,^88^ and supercapacitors.^46,149,150^ HESs offer significant potential for electrochemical energy storage due to their unique multi-cation compositions, which provide enhanced structural stability, high electronic conductivity, and improved ion transport properties. Meanwhile, the ability to fine-tune the composition and defect structures of HESs allows for the optimization of their electrochemical performance.

**Table 1:** A comparison of the electrochemical performance of HES anodes in LIBs

Composition	Synthesis method	Initial capacities (mA h g^−1^)@current density	Cycle retention (cycles)@current density	Rate capability (mA h g^−1^)@current density	Ref.
(MgTiZnCuFe)_3_O_4_	Solid-state	634@100 mA g^−1^	79% (300)@0.1 A g^−1^	272@2 A g^−1^	34
(NiCoMnFeTi)_3_O_4_	Solid-state	560@100 mA g^−1^	100% (100)@0.1 A g^−1^	343@2.5 A g^−1^	109
(FeCoNiCrMn)_3_O_4_	Hydrothermal	1235@20 mA g^−1^	90% (200)@0.5 A g^−1^	500@2 A g^−1^	143
(FeCoNiCrMn)_3_O_4_	Solid-state	680@100 mA g^−1^	60% (300)@0.5 A g^−1^	182@2 A g^−1^	45
(FeCoNiCrMnZnLi)_3_O_4_	Solid-state	1050@50 mA g^−1^	50% (100)@0.5 A g^−1^	200@2 A g^−1^	144
(Al_0.2_FeCoNiCrMn)_3_O_4_	Solid-state	1400@200 mA g^−1^	40% (500)@0.2 A g^−1^	634@3 A g^−1^	77
(CoCrFeMnNi)_3_O_4_	SCS	1133@100 mA g^−1^	84% (200)@1 A g^−1^	48@10 A g^−1^	78
Sn_0.8_(Co_0.2_Mg_0.2_Mn_0.2_Ni_0.2_Zn_0.2_)_2.2_O_4_	Solid-state	600@50 mA g^−1^	100% (500)@0.2 A g^−1^	182@1 A g^−1^	145
(CrNiMnFeCu)_3_O_4_	Hydrothermal	800@50 mA g^−1^	100% (400)@0.5 A g^−1^	480@2 A g^−1^	108
(CrMnFeNiCu)_3_O_4_	Hydrothermal	750@50 mA g^−1^	100% (150)@0.5 A g^−1^	340@2 A g^−1^	113
(CrMnFeNiCu)_3_O_4_	Hydrothermal	755@50 mA g^−1^	99% (250)@0.5 A g^−1^	451@2 A g^−1^	75
(FeCoNiCrMn)_3_O_4_	Solid-state	1645@100 mA g^−1^	86% (1200)@2 A g^−1^	596@2 A g^−1^	141
Li_1.8_(FeCoZnCrMn)_3_O_*x*_	Sol–gel	733@20 mA g^−1^	81% (300)@0.5 A g^−1^	146@1 A g^−1^	87
(CrMnFeNiZn)_3_O_4_ (C + T)	Solvothermal	865@50 mA g^−1^	90% (200)@0.5 A g^−1^	560@3 A g^−1^	101
(ZnCoMnFeAlMg)_3_O_4_	Coprecipitation	950@1000 mA g^−1^	81% (5000)@2 A g^−1^	300@2 A g^−1^	146
(MnFeCoNiZn)_3_O_4_	Electrospinning	1284@20 mA g^−1^	100 (550)@0.5 A g^−1^	58@2 A g^−1^	89
(FeCoNiCrZn)_3_O_4_	Sol–gel	1152@200 mA g^−1^	99% (1000)@1 A g^−1^	220@30 A g^−1^	147
(FeCoNiCuZnMn)_3_O_4_	Solid-state	740@100 mA g^−1^	86% (100)@0.13 A g^−1^	300@1.6 A g^−1^	84
(CoMnZnNiMg)_2_CrO_4_	SCS	1483@200 mA g^−1^	95% (200)@0.2 A g^−1^	371@2 A g^−1^	79
(FeCoCrMnZn)_3_O_4_	Solid-state	477@200 mA g^−1^	95% (2000)@0.2 A g^−1^	829@2 A g^−1^	142
(CrFeMnNiCo_3_)_3_O_4_	SCS	506@200 mA g^−1^	100% (800)@0.2 A g^−1^	147@2 A g^−1^	148
(MgCoNiCuZn)Fe_2_O_4_	Solid-state	812@50 mA g^−1^	80% (650)@1 A g^−1^	87@10 A g^−1^	71
(LiFeNiMnCuZn)_3_O_4_	Laser-based	866@500 A g^−1^	100% (800)@0.5 A g^−1^	585@2 A g^−1^	85

#### 3.2.4 Other electrocatalytic applications

HESs have also shown promise in other electrocatalytic reactions, including organic compound oxidation, the ammonia oxidation reaction (AOR), and the nitrate reduction reaction (NO_3_^−^RR).^22,47,151^ For example, Gu *et al.* synthesized defect-rich HES ((FeCrCoNiCu)_3_O_4_) nanosheets *via* a low-temperature plasma method and used them for 5-hydroxymethylfurfural (HMF) electrooxidation for the first time.^47^ Benefiting from high surface area and abundant oxygen vacancies, the (FeCrCoNiCu)_3_O_4_) nanosheets exhibited higher activity for HMF oxidation, characterized by a lower onset potential and faster kinetics, in comparison to HESs synthesized by a reverse co-precipitation method at 1000 °C.

**Fig. 11:**
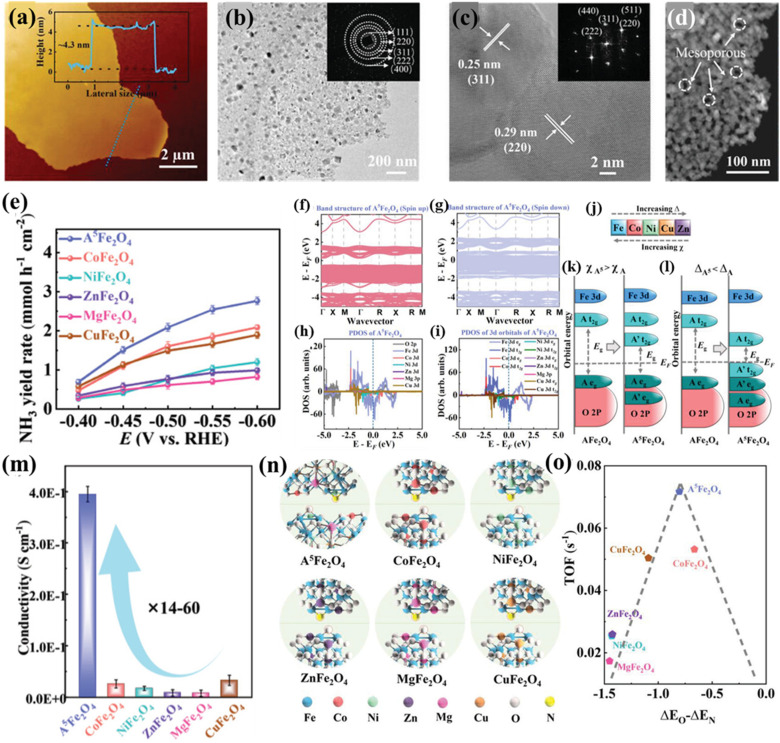
HESs for the electrochemical NO_3_^−^RR. (a) Thickness measurement of (Co_0.2_Ni_0.2_Zn_0.2_Mg_0.2_Cu_0.2_)Fe_2_O_4_ (A^5^Fe_2_O_4_) nanosheets by AFM, (b) TEM (the inset shows the SAED patterns), (c) HRTEM, and (d) HAADF-STEM image of A^5^Fe_2_O_4_, (e) NH_3_ yield rate of A^5^Fe_2_O_4_ and binary spinel oxides at different potentials, the calculated band structure of A^5^Fe_2_O_4_ (f) (spin up) and (g) (spin down), (h and i) PDOS for A^5^Fe_2_O_4_, (j) trends in crystal field splitting *Δ* and electronegativity *χ* for the five metals of the HES, schematic illustration that (k) adding a transition metal with greater electronegativity reduced the bandgap energy by introducing states between the e_g_ and t_2g_ states of the original transition metal and (l) adding a transition metal of lower crystal field splitting reduced the bandgap energy both by introducing new occupied t_2g_ states above the e_g_ states of the original transition metal and by lowering its unoccupied t_2g_ states, (m) electron conductivity of all spinel oxides, (n) optimized structural model of adsorbate nitrogenous compounds (*NO, *N, *NH, *NH_2_, and *NH_3_) and oxycompounds (*NO_3_, *NO_2_, and *OH) on A^5^Fe_2_O_4_ and other binary spinels, and (o) a volcano-type relationship between the binding difference of O and N (Δ*E*_O_ − Δ*E*_N_) and TOF values of different spinel catalysts. Reproduced with permission.^22^ Copyright 2022, John Wiley and Sons.

Qi *et al.* designed ultrathin high-entropy Fe-based spinel oxide (Co_0.2_Ni_0.2_Zn_0.2_Mg_0.2_Cu_0.2_)Fe_2_O_4_ (A^5^Fe_2_O_4_) nanosheets for the NO_3_^−^RR to NH_3_.^22^ A^5^Fe_2_O_4_ had a thickness of only 4.3 nm (Fig. 11a) investigated by atomic force microscopy (AFM) with a spinel structure (Fig. 11b and c) and abundant mesoporous structures between two interconnected nanoparticles (Fig. 11d). These A^5^Fe_2_O_4_ nanosheets showed excellent performance for the NO_3_^−^RR with an NH_3_ yield rate of ≈2.1 mmol h^−1^ cm^−2^ at −0.5 V *versus* the reversible hydrogen electrode, outperforming other binary Fe-based spinels (AFe_2_O_4_), as shown in Fig. 11e. DFT calculations suggested that introducing multicomponents could significantly narrow the bandgap and increase states around the Fermi level, achieving the transformation of spinel oxides from semiconductors into metalloids (Fig. 11f and g). The doped transition metals (Co, Ni, Zn, and Cu) with lower crystal field splitting and higher electronegativity could influence the bandgap by lowering the energy of the unoccupied t_2g_ states of B metals and introduce a new state within the bandgap of A metals, which made all metals lose their own electronic properties and become degenerated (Fig. 11j–l). The narrower bandgap of A^5^Fe_2_O_4_ was also confirmed by diffuse reflectance spectra (DRS) measurements, which endowed A^5^Fe_2_O_4_ with a high electron conductivity, more than an order of magnitude higher than that of AFe_2_O_4_ (Fig. 11m). In addition, the density of states (DOS) of A^5^Fe_2_O_4_ near the Fermi level was mainly contributed by d-orbitals of Fe (Fig. 11h and i), indicating that the electrocatalytic activity of the HES mainly originated from Fe sites. The lower surface work function (WF) of A^5^Fe_2_O_4_ than that of AFe_2_O_4_ indicated that electrons could more easily transfer from the catalyst surface to the adsorbed reaction intermediates, thereby promoting catalytic reaction kinetics. Mechanistic investigations by experimental and computational methods revealed that constructing high-entropy A^5^Fe_2_O_4_ could adjust the adsorption strength of NO_3_^−^ and other reaction intermediates for boosting the NO_3_^−^RR (Fig. 11n and o).

### 3.3 Photocatalysis and advanced oxidation

**Fig. 12:**
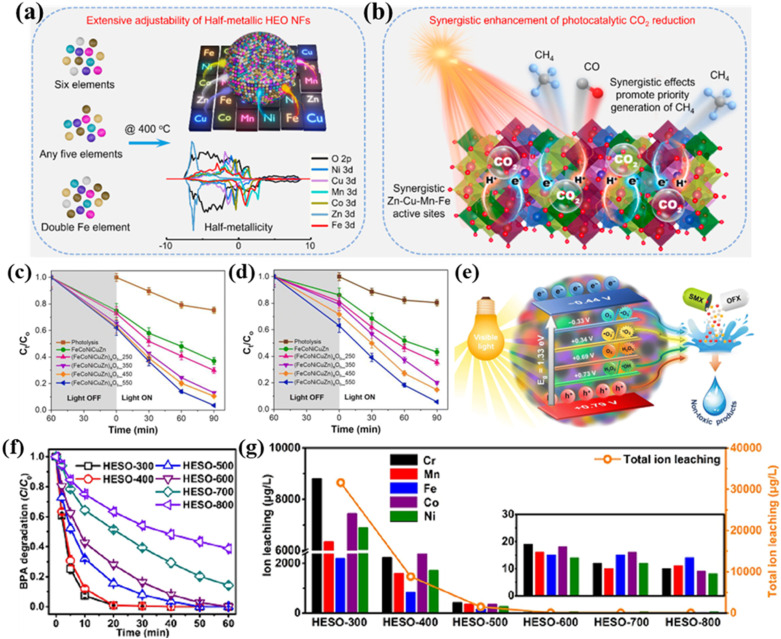
HESs for photocatalysis and AOPs. (a) Structurally adjustable HES nanofibers with half metallicity, and (b) schematic illustration of the synergistic activation and conversion of CO_2_ into CH_4_ of the HES nanofibers. Reproduced with permission.^82^ Copyright 2024, John Wiley and Sons. Visible light-induced photocatalytic degradation of (c) SMX and (d) OFX by FeCoNiCuZn and (FeCoNiCuZn)_*a*_O_*b*_, and (e) schematic illustration of the photocatalytic degradation mechanism of (FeCoNiCuZn)_*a*_O_*b*_ under visible light irradiation.^152^ (f) BPA degradation efficiency and (g) the concentration of total leached metal ions in PMS-AOPs systems with (Cr_0.2_Mn_0.2_Fe_0.2_Co_0.2_Ni_0.2_)_3_O_4_ (HESO) calcined at different temperatures as catalysts. Reproduced with permission.^153^ Copyright 2023, Elsevier.

Despite being less studied in the area of photocatalysis, HESs have the ability to arrange various components and tune properties to achieve a photoresponse, which has attracted increasing attention in photocatalysis in recent years. For example, Zhang *et al.* explored the photocatalytic potential of porous HES nanofibers (NFs) of (NiCuMnCoZnFe)_3_O_4_ for CO_2_ reduction.^82^ A series of characterization studies showed that the as-prepared HES NFs had a high surface area of 66.48 m^2^ g^−1^, abundant lattice strains and defects, a dual bandgap, and semi-metallic properties. The dual-bandgap structure and half-metallicity endowed them with remarkable light-absorbing capacities (Fig. 12a). *In situ* FTIR results show that the incorporation of multiple elements created a wide range of synergistic catalytic sites capturing intermediates and protons, and oxygen vacancies can serve as active sites for electron–hole separation and CO_2_ adsorption and help break C=O bonds to produce CO (Fig. 12b). All of these made the HES NFs excellent photocatalysts to efficiently convert CO_2_ into CH_4_ and CO with high yields of 8.03 and 15.89 μmol g^−1^ h^−1^, respectively, without using photosensitizers or sacrificial agents.

Das *et al.* applied HESs (FeCoNiCuZn)_*a*_O_*b*_ for photocatalytic degradation of organic pollutants in water and wastewater treatment processes.^152^ As shown in Fig. 12c and d, the (FeCoNiCuZn)_*a*_O_*b*_ nanoparticles exhibited a good photocatalytic degradation efficiency of above 95% for two model antibiotics (*i.e.*, sulfamethoxazole and ofloxacin) under visible light irradiation for 90 min without any significant metal leaching. This outstanding photocatalytic performance can be mainly attributed to the narrow bandgaps, reduced electron–hole recombination and thus extended carrier-lifetime, and enhanced electronic conductivity (Fig. 12e). In addition, the even distribution of metal cations with different oxidation states in HESs promoted the activation of lattice oxygen in the form of surface-confined oxygen vacancies and the formation of reactive species for oxidation reactions.

In addition to the catalytic applications above, HESs have also been explored as catalysts in advanced oxidation processes (AOPs) for organic pollutant degradation. Wang *et al.* demonstrated that spherical mesoporous HESs (*e.g.*, Ni–Co–Cr–Fe–Mn spinel oxide) could effectively activate PMS for degradation of methylene blue (MB).^67^ Nearly 95% of MB was degraded after 60 min with a total organic carbon removal of 71.7%. Radical scavenging experiments indicated that both SO_4_˙^−^ and HO˙ were involved in the oxidation and SO_4_˙^−^ played a dominant role. After the reaction, the concentrations of leached metal ions (Ni, Co, Cr, Fe, and Mn) in the treated solution were very low, indicating the structural robustness of HESs in oxidation reactions. Zhang *et al.* successfully prepared an entropy-stabilized HES ((Cr_0.2_Mn_0.2_Fe_0.2_Co_0.2_Ni_0.2_)_3_O_4_) as a PMS catalyst for bisphenol A (BPA) degradation.^153^ As shown in Fig. 12f and g, the HES calcined at 600 °C achieved complete degradation of BPA within 60 min and also exhibited good structural and chemical stability, which had a minimal total metal ion leaching (82 μg L^−1^) and maintained an efficiency of over 90% even after 5 cycles. XPS analysis before and after the reaction indicated that Co(ii) was the dominant active site for PMS activation to generate radicals (HO˙ and SO_4_˙^−^) for rapid BPA degradation. The presence of oxygen vacancies could contribute to the generation of reactive oxygen species and enhance the catalytic performance of catalysts. DFT calculations suggested that the adsorption energy of PMS molecules on the Co sites in the HES was more negative than that of a binary spinel ((Co_0.2_Fe_0.8_)_3_O_4_), indicating that the electronic structures of Co in HES were optimized by its disorder arrangement of metal sites and defect structure, thus promoting electron transfer between the Co sites and PMS. Due to the acidic nature of PMS, it is crucial to develop stable HESs without severe metal leaching during reactions to avoid secondary pollution.

## 4. Discussion

**Fig. 13:**
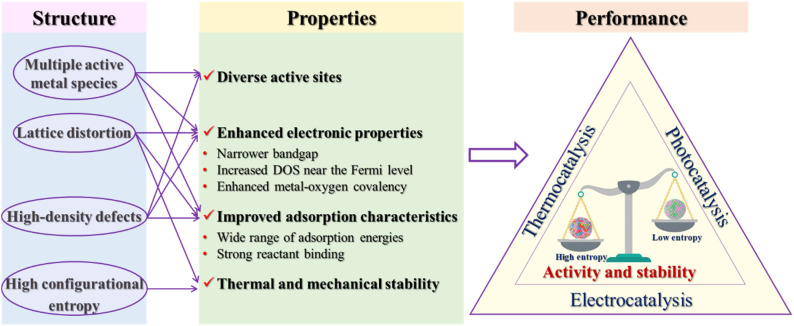
Relationships between the structure, properties and catalytic performance of HESs.

The diverse catalytic applications of HESs, as summarized above, highlight their potential in thermocatalysis, electrocatalysis, photocatalysis and AOPs. While catalytic mechanisms of HESs have also been investigated for each reaction, variations across studies have been observed. As research on HESs is currently in its nascent stages, further efforts are still required to reveal the complex structure–property–performance relationships of HESs in various catalytic processes. The compositional and structural diversity of HESs makes it extremely challenging to reveal the mechanisms underlying their enhanced catalytic performance compared with low-entropy spinels or other catalysts. Here, we summarize the strategies for fine-tuning the properties of HESs arising from the flexible high-entropy structure to benefit the catalytic performance of HESs as shown in Fig. 13. Key structural characteristics of HESs including multiple active metal species, lattice distortion, high-density defects, and high configurational entropy lead to the optimization of critical catalytic properties such as active site diversity, electronic properties, adsorption characteristics, and stability. These properties collectively enhance the catalytic performance of HESs in comparison to lower entropy materials.

In heterogeneous catalysis, active sites are responsible for reactant adsorption, bond breaking and forming, interfacial charge transfer, intermediate stabilization, and product production and desorption. For metal-based catalysis, multiple active metal cations with various valence states in HESs can provide more active sites than traditional spinels.^127^ Rather than merely reflecting the average activity of each metal site, these elements demonstrate significantly higher activity due to the collaborative and synergistic effects resulting from their homogeneous distribution and inter-element interactions.^40,106^ As a result, the multiple metal cations in HES can provide favorable adsorption sites for different reactants/intermediates and pathways, thus facilitating the overall catalytic performances.^154^ Several studies suggested that there is no simple linear trend between the molar ratio of each element and the related catalytic activity, confirming the synergy between multiple metal cations in HESs.^48,155^

In addition, high-density defects in HESs can also act as active sites, contributing to the excellent catalytic performance of HESs. In HES phases, the diverse bonding configurations between various metal ions and oxygen atoms generate a disordered electronic environment around the oxygen atoms, which will induce the detachment of these ions to form defects.^156^ These defect structures include point defects (*e.g.*, vacancies and interstitials), line defects (*e.g.*, dislocations), and interfaces (*e.g.*, grain boundaries and surfaces).^10,68,157^ In particular, oxygen vacancies have been demonstrated to influence the catalytic activity by providing additional catalytic sites, modifying electronic properties of surrounding metals, and affecting the adsorption configuration of reaction intermediates, which have attracted the most interest in the high entropy community to improve the catalytic performance of HESs.^21,78,108,122,158–163^

The electronic structure of catalysts is considered a critical factor in regulating electron transfer processes and determining the binding strength between catalysts and adsorbates.^164^ An explicit electronic structure analysis could provide important clues for understanding the enhanced catalytic performance of HESs. In transition metal oxides, the d-electrons of transition metals play a major role in shaping their electronic structures. The presence of various metal elements in HESs leads to an extremely complex electronic landscape with new electronic states that may facilitate catalytic reactions.^15,23,49,165,166^ More importantly, high entropy configuration induces significant lattice distortion and defects in spinels, fundamentally altering the electronic properties of spinels, such as the band structure, DOS, the distribution of electronic states, and e_g_ orbital occupancy.^16,22,45,82,137,163,167,168^ For example, Katzbaer *et al.* demonstrated band gap narrowing in a high-entropy aluminate spinel oxide (Fe_0.2_Co_0.2_Ni_0.2_Cu_0.2_Zn_0.2_)Al_2_O_4_ with a 0.9 eV band gap, significantly narrower than the band gaps of its single-metal end members (ranging from 1.6 to 4.2 eV).^14^ First-principles calculations showed that the narrower band gap originated from the wider energy distribution of the 3d states due to the different electronegativities and crystal field splitting energies between 3d transition metals. Tian *et al.* found that the Co–O octahedron in spinel undergoes asymmetric distortion in a high-entropy atomic environment, resulting in the redistribution of internal charges in the Co–O octahedron.^23^ Near the Fermi level, the DOS of HESs ((Co_0.2_Mn_0.2_Ni_0.2_Fe_0.2_Cr_0.2_)_3_O_4_) was obviously higher than that of Co_3_O_4_ and (Co_0.25_Ni_0.25_Fe_0.25_Mn_0.25_)_3_O_4_, which means the highest conductivity of HESs among the three materials. The difference between the O p band center and the Co d band center in HESs was also smaller, indicating stronger Co–O covalency. More importantly, the overall d band center of HES had an upward shift closer to the Fermi level, which is favorable for the strong adsorption of reaction intermediate on the HES surface. Adsorption energy of reactive intermediates on the catalyst surface is one of the most important descriptors for catalytic reactions, which successfully bridge the gap between a catalyst's structure and activity through the Sabatier principle and the d-band theory.^169^ DFT calculations in many studies have indicated that HESs can provide access to a broad range of adsorption energies which could be optimum for the intermediates due to their diverse components and configurations.^21,132,170^

One of the primary benefits of high-entropy engineering for spinels is structural stabilization. The spinel structure provides not one but two cation sublattices: a tetrahedrally coordinated A site forming a diamond lattice and an octahedrally coordinated B site forming a pyrochlore lattice.^18^ A significant increase in configurational entropy can be anticipated when both sublattices are occupied by a mixture of cations.^57,82,97,171^ Due to the high-entropy effect, HESs obtain lower formation energy of the spinel structure and increase its thermodynamic stability, especially at high temperatures. Kinetically, high-entropy mixing may also enhance structural stability due to lattice distortion, which can create significant diffusion barriers that help prevent phase segregation, particularly at low temperatures. This stability is crucial for maintaining the integrity of the catalysts under harsh reaction conditions, as evidenced by their consistent performance in high-temperature and electrochemical catalytic reactions.^143,172^ For example, Han *et al.* reported that the high-entropy effect stabilized the spinel structure of (Fe_0.2_Mg_0.2_Mn_0.1_Al_0.3_Cr_0.2_)_3_O_4_ at 900 °C even with a large amount of oxygen converted and thus promoted the exsolution and stabilization of substantial Fe^0^, which resulted in extensive metal–oxide interfaces with metal–oxygen vacancy pairs responsible for the efficient and stable solar thermochemical water splitting.^110^

## 5. Summary and outlook

HESs have emerged as a typical representative of HEMs, offering more possibilities for future energy and environmental materials. In this review, the recent advances in applying HESs for heterogeneous catalysis are presented, including the unique features, synthesis, characterization, and catalytic applications. From the wide range of studies, the underlying relationships among the special structure, favorable catalytic properties, and performance improvement of HESs in catalysis are summarized and discussed. Although significant progress has been made in the study of HESs, further efforts are required in several areas, including precise synthesis, advanced characterization, and expanded applications, to fully unlock the potential of HESs in catalysis. Notably, despite efforts made to elucidate the structure–property–performance relationships of HESs, a comprehensive and deep understanding is still lacking due to the compositional and structural complexity of HESs. Many studies commonly attributed the excellent performance of HESs in catalytic reactions to the presence of oxygen vacancies and/or a synergistic effect, but it remains unclear how oxygen vacancies specifically impact the performance of HESs in a catalytic reaction and what the exact nature of the synergistic effect is. More importantly, accurate identification of active sites is crucial for the design, synthesis, modification, and enhancement of metal-based catalysts. For HESs, the vast number of potential surface atom arrangements makes it challenging to identify the actual active sites by studying only one or a few models. This requires an integrated approach combining experimental and computational studies to provide insights into the structure and properties directly related to catalytic performance of HESs and help elucidate the catalytic reaction mechanisms, optimizing their performance for various catalytic applications.

The development of advanced *in situ*/*operando* characterization techniques will offer valuable insight into the fundamental understanding of active sites and reaction pathways in HESs for catalysis. For instance, *in situ* TEM allows real-time tracking of phase transformations induced by catalytic reactions and morphological changes of individual HES nanoparticles.^173^ By analyzing *in situ* electron diffraction (ED) data along with the spatiotemporal changes in valence states of metal species, the formation sequences of distinct phases can be clearly identified. This provides direct insights into the conversion and reaction kinetics, thereby enhancing our understanding of the dynamic catalytic mechanisms. *Operando* XAS is another powerful tool for revealing the interactions among elements within a high-entropy phase and the dynamic interplay between active sites and reactants, intermediates, or products during a reaction.^174^ However, for complex systems such as HESs, “trial and error” experiments are low-efficiency and time-consuming, while conventional theoretical simulations involve high computational costs and limited accuracy. With the fast development of computational chemistry, artificial intelligence (AI) and machine learning (ML) have revolutionized the field of materials science by enabling the prediction of properties, optimization of compositions, and discovery of new materials. For example, ML regression can be trained on experimental data to predict properties based on compositional and structural features and optimize the composition of catalysts to achieve desired properties by exploring a vast compositional space efficiently. In addition, high-throughput experimental methods, enabled by the advancement in instrumentation and electronics, will speed up the generation of extensive results and promote the identification of desired products, thereby enriching knowledge in computational design. It can be envisioned that these data-driven methodologies will unveil the complexities of HESs and advance the field toward the rational design of robust HESs with enhanced properties for catalytic applications.

While this review has focused on HESs, the underlying concepts can be applicable to other HEMs as well. We anticipate that the exploration of HESs in catalysis will innovate traditional paradigms of material design, promoting a multidisciplinary approach that integrates materials science, chemical engineering, sustainable technology, and computational chemistry. Advancements in this field could revolutionise the development of new and complex catalysts, moving beyond the limitations of conventional transition metals and oxides to achieve greater efficiency, stability, and environmental compatibility. This progress could provide more effective solutions for tackling global challenges in energy and sustainability.

## Data availability

No primary research results, software or code have been included and no new data were generated or analyzed as part of this review.

## Author contributions

The manuscript was written by Y. L. Mo. X. G. Duan and S. B. Wang supervised the work and contributed to revising and refining the manuscript. X. H. Guan provided valuable suggestions for the manuscript.

## Conflicts of interest

There are no conflicts to declare.
